# Supplementary material for: Spatial-linked alignment tool (SLAT) for aligning heterogenous slices
Source: Nat Commun. 2023 Nov 9;14:7236. doi: 10.1038/s41467-023-43105-5 (PMC10636043; doi:10.1038/s41467-023-43105-5)
Supplement: Supplementary file 1 — Supplementary Information [file 41467_2023_43105_MOESM1_ESM.pdf]

Spatial-linked alignment tool (SLAT) for aligning heterogenous slices

Chen-Rui Xia[1,2†], Zhi-Jie Cao[1,2†*], Xin-Ming Tu[1,#], and Ge Gao[1,2*]

[1] State Key Laboratory of Protein and Plant Gene Research, School of Life Sciences, Biomedical Pioneering Innovative Center (BIOPIC) and Beijing Advanced Innovation Center for Genomics (ICG), Center for Bioinformatics (CBI), Peking University, Beijing 100871, China

[2] Changping Laboratory, Beijing 102206, China.

[†] These authors contributed equally to this work.

- Authors to whom correspondence should be addressed: gaog@mail.cbi.pku.edu.cn (for G.G.), or caozj@mail.cbi.pku.edu.cn (for Z.J.C)

[#] Current address: Paul Allen School of Computer Science and Engineering, University of Washington, Seattle, WA 98195, United States

This Supplementary Information file contains:

Supplementary Figures 1-30
Supplementary Tables 1-3

Supplementary Figures

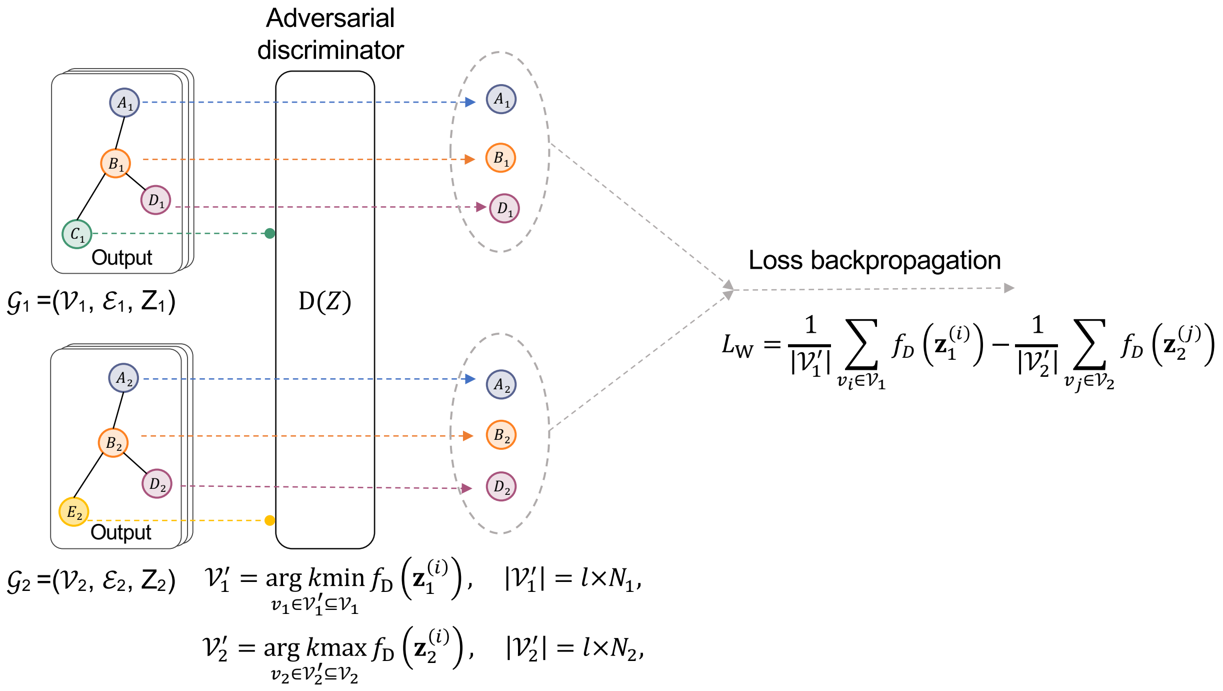

Supplementary Fig. 1 Illustration of dynamic clipping.
Illustration of the dynamic clipping criteria used to select cells for adversarial training. Only a subset of cells from the two datasets with minimal Wasserstein distance are selected (see **Methods**).

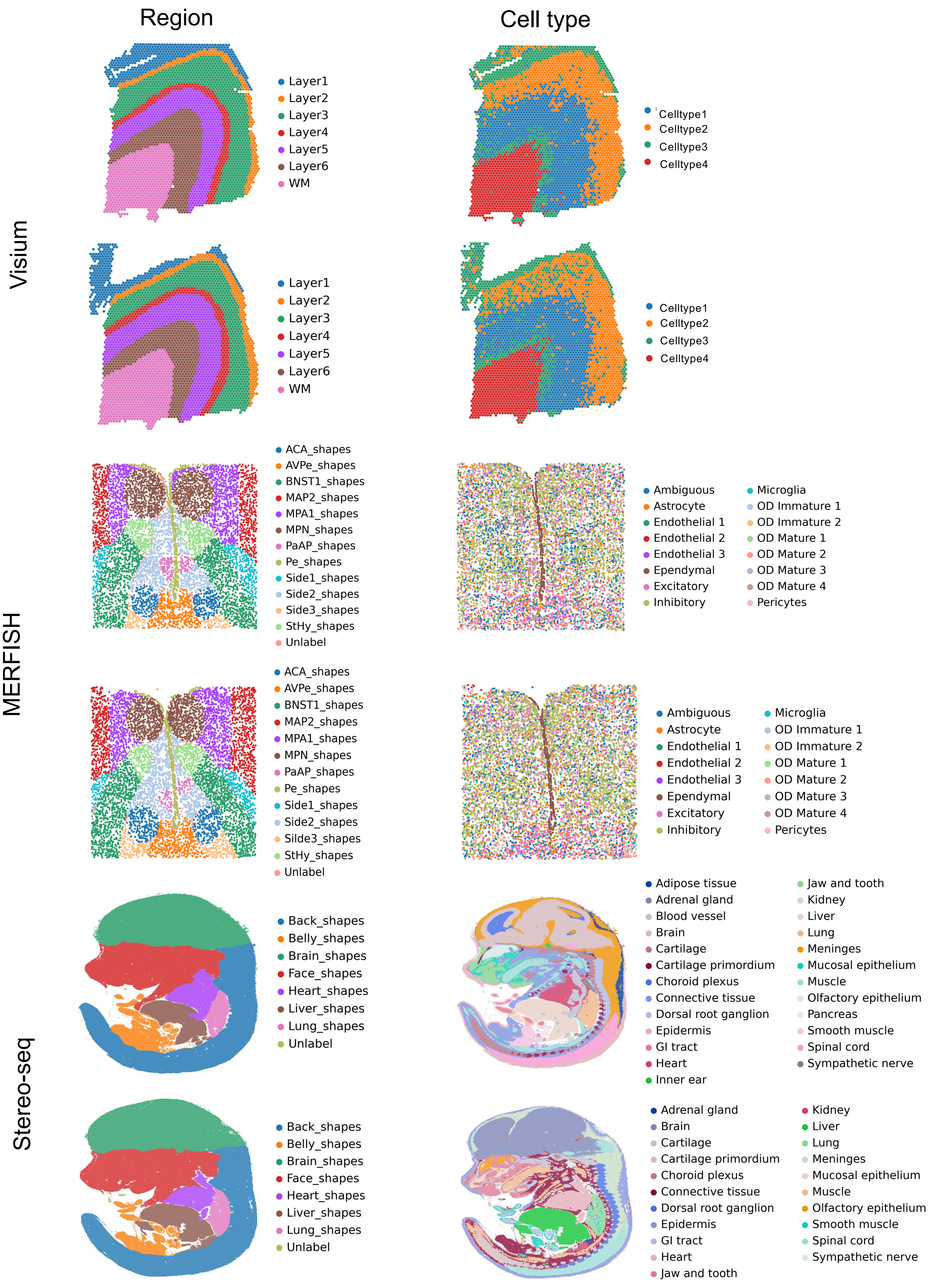

Supplementary Fig. 2 Visualization of the first two slices of benchmark datasets. Visualization of slices used in the Fig. 2. Slices are colored by spatial regions (left panels) and cell types (right panels), respectively.

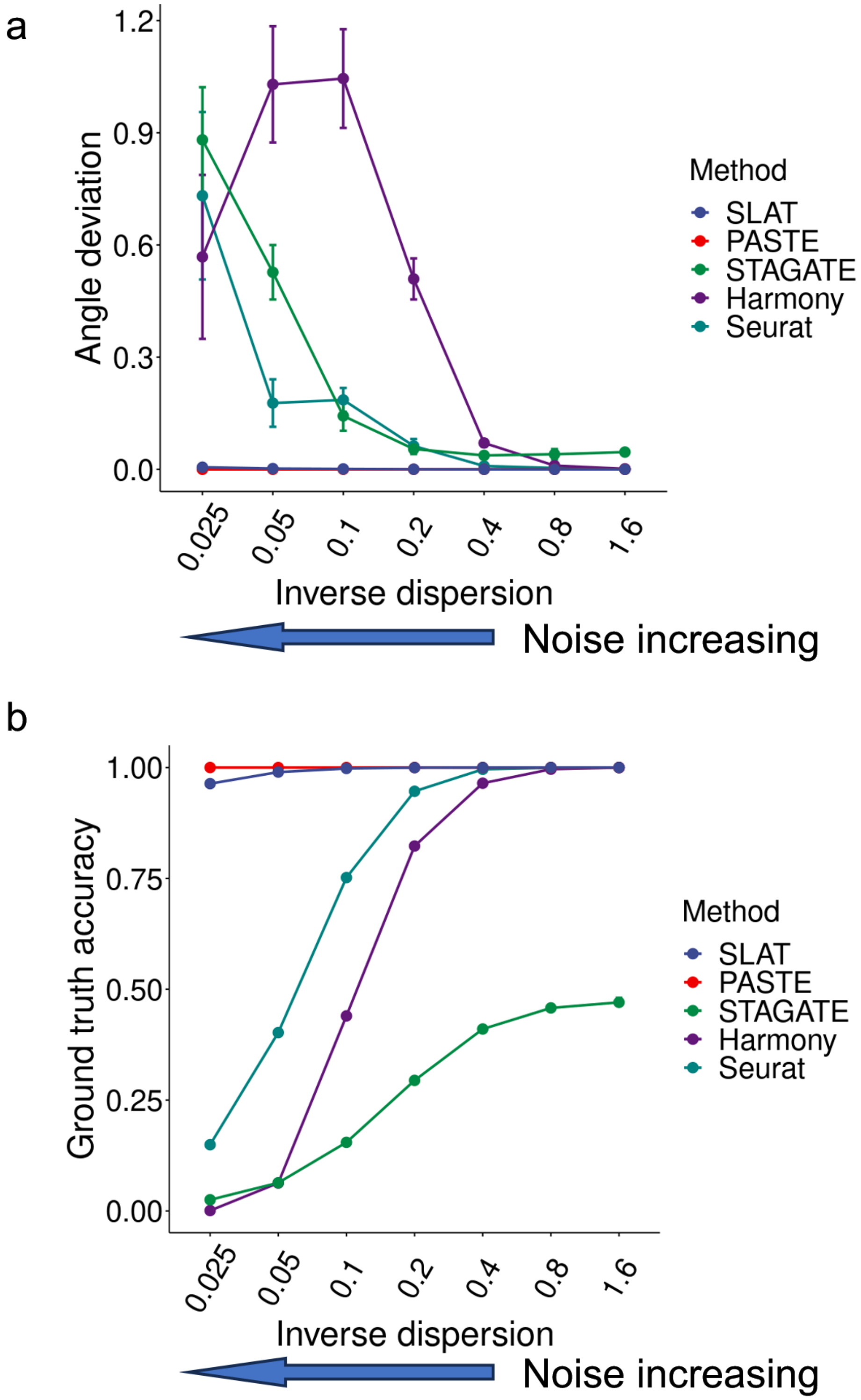

Supplementary Fig. 3 Benchmark in rotated and perturbed duplicated slice alignment.

a, Deviation of the estimated corrective rotation angle compared to ground truth. **b**, Ground truth accuracy in duplicated slice alignment. We noticed that PASTE always produces the perfect matching even at very high noise levels, which is possibly due to the fact that duplicated slices share the exact same shape, which forms an unmistakable optimum in Gromov-Wasserstein transport of spatial coordinates. n = 8 repeats with different sampling random seeds. Error bars indicate mean ± s.d. From right to left, Smaller inverse dispersion indicates higher noise level. Source data are provided as a Source Data file.

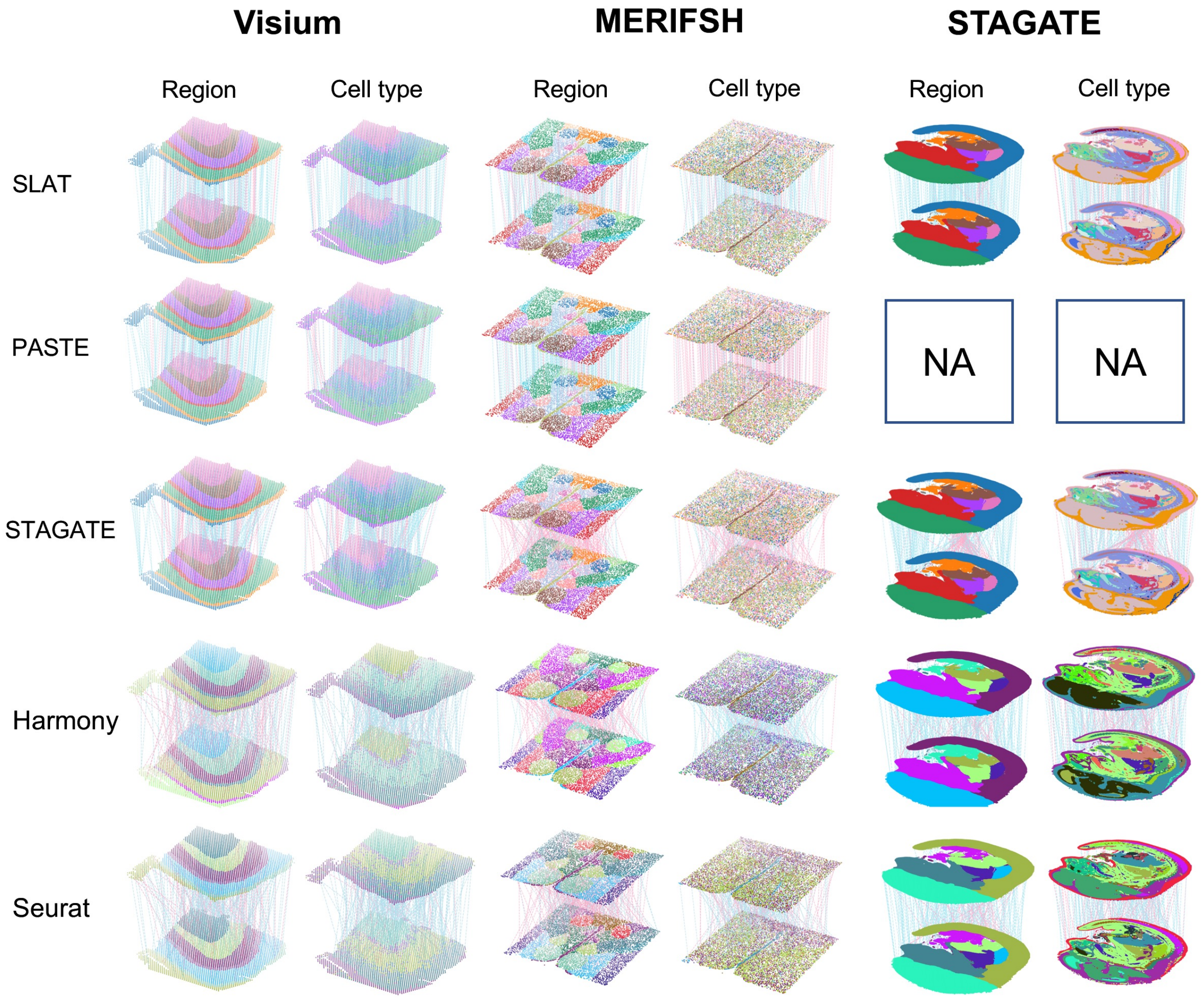

Supplementary Fig. 4 Visualization of evaluation SLAT on homogeneous spatial alignment with spatially unaware algorithms.
Visualization of alignment results of different methods on the benchmark datasets in Fig. 2a. Vertical lines connect aligned cell pairs (subsampled to 300 alignment pairs for clear visualization). Blue lines indicate correct alignments (with matching cell types and spatial regions) while red lines indicate incorrect alignments.

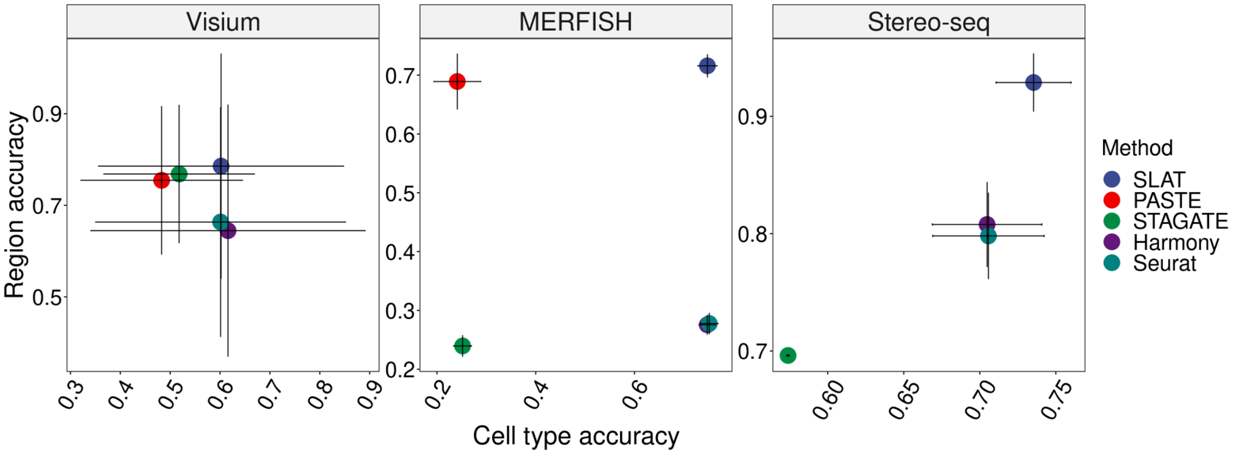

Supplementary Fig. 5 Aggregated metrics for all slices.

Aggregated cell-type matching accuracy (y-axis) and region-matching accuracy (x-axis) in all slices. Source data are provided as a Source Data file.

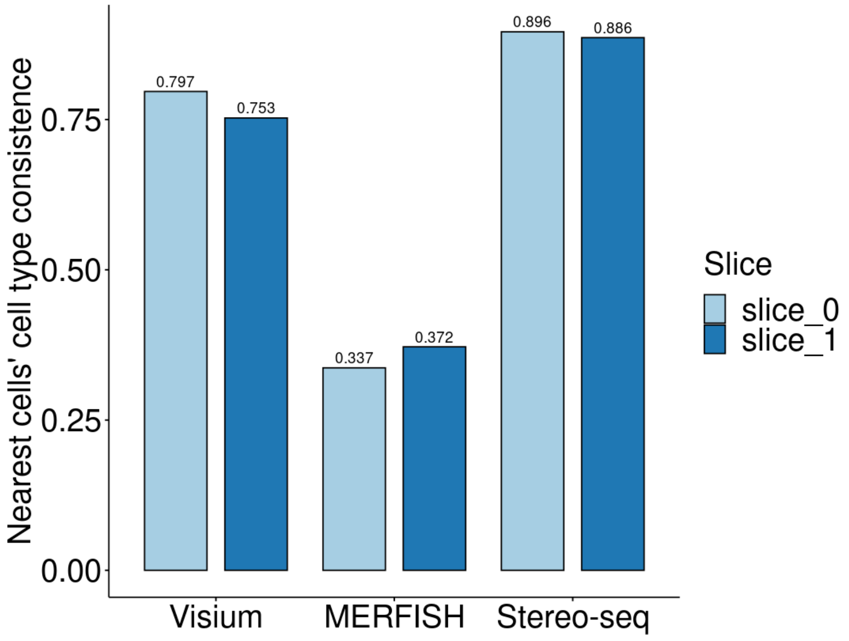

Supplementary Fig. 6 Spatial neighbors' cell type consistence.
Ratio of neighbor cells that belong to same cell type, which measures the correlation between cell type and spatial region. The two slices of each technology are shown separately. Source data are provided as a Source Data file.

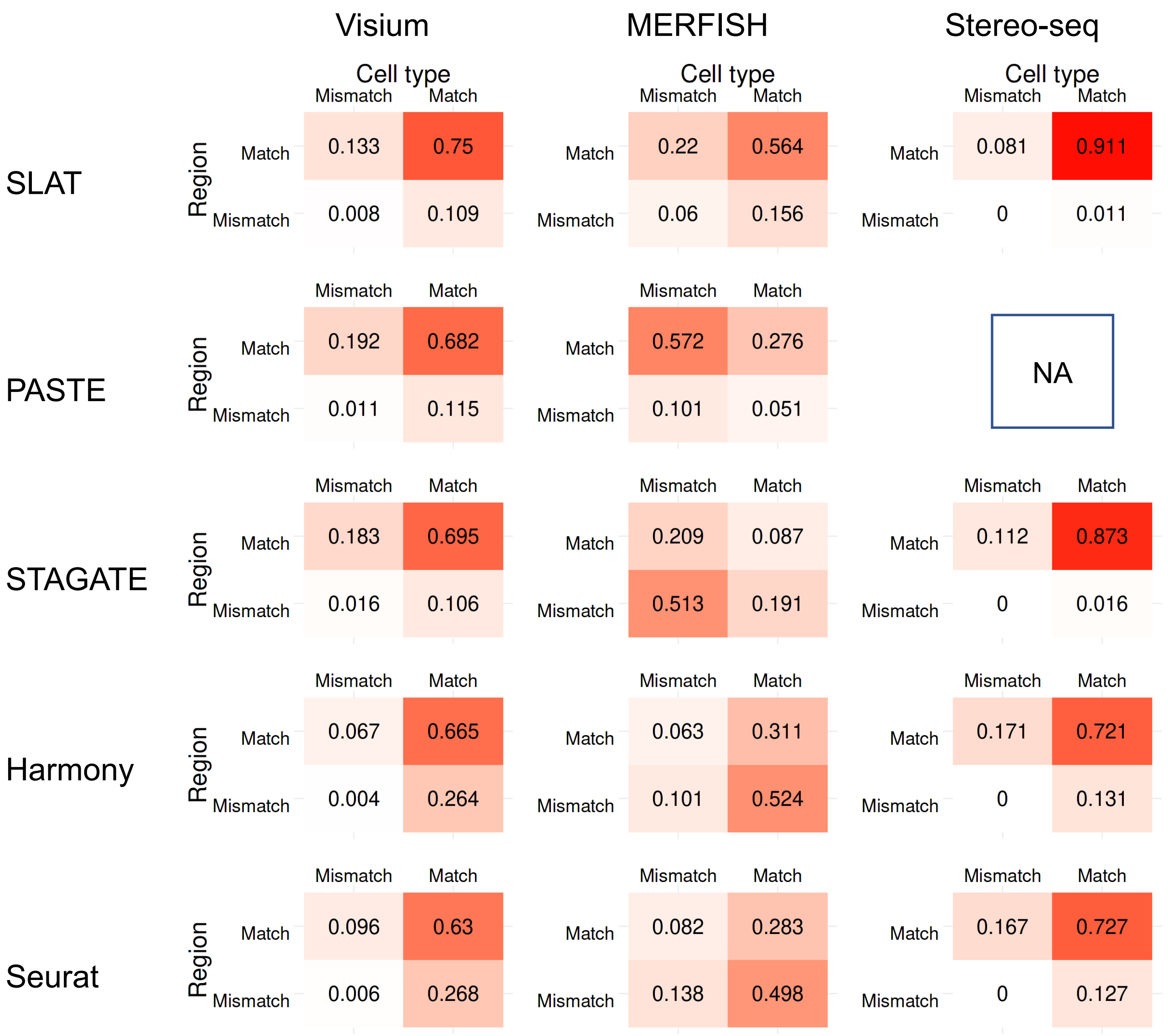

Supplementary Fig. 7 Evaluation of different methods on split spatial datasets. Heatmaps quantifying the region matching accuracy and cell type matching accuracy of SLAT, PASTE, STAGATE, Harmony and Seurat respectively in the form of contingency tables of split datasets. The number in each cell is the average proportion across eight repeats with different random seeds. PASTE failed to run on the Stereo-seq dataset due to GPU memory overflow (capping at 80 GB). Source data are provided as a Source Data file.

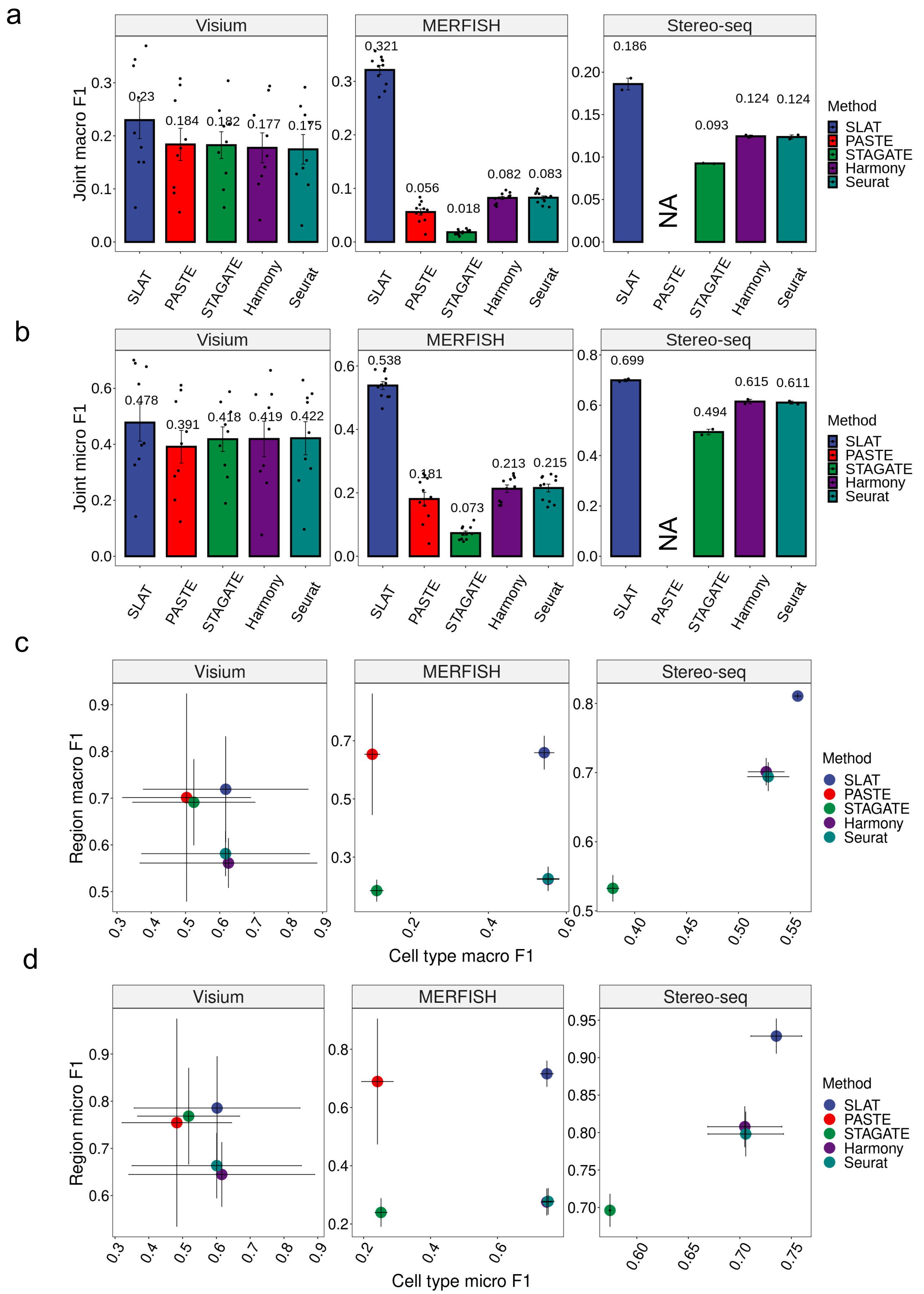

Supplementary Fig. 8 Aggregated macro and micro F1 metrics.

a, Joint macro F1 metric. **b**, Joint micro F1 metric. **c,** Macro F1 metric for cell type matching accuracy (x-axis) and region matching accuracy (y-axis). **d**, Micro F1 metric for cell type matching accuracy (x-axis) and region matching accuracy (y-axis). *n* = 9,11 and 2 for Visium, MERFISH and Stereo-seq datasets, respectively. Error bars indicate mean ± s.d. Source data are provided as a Source Data file.

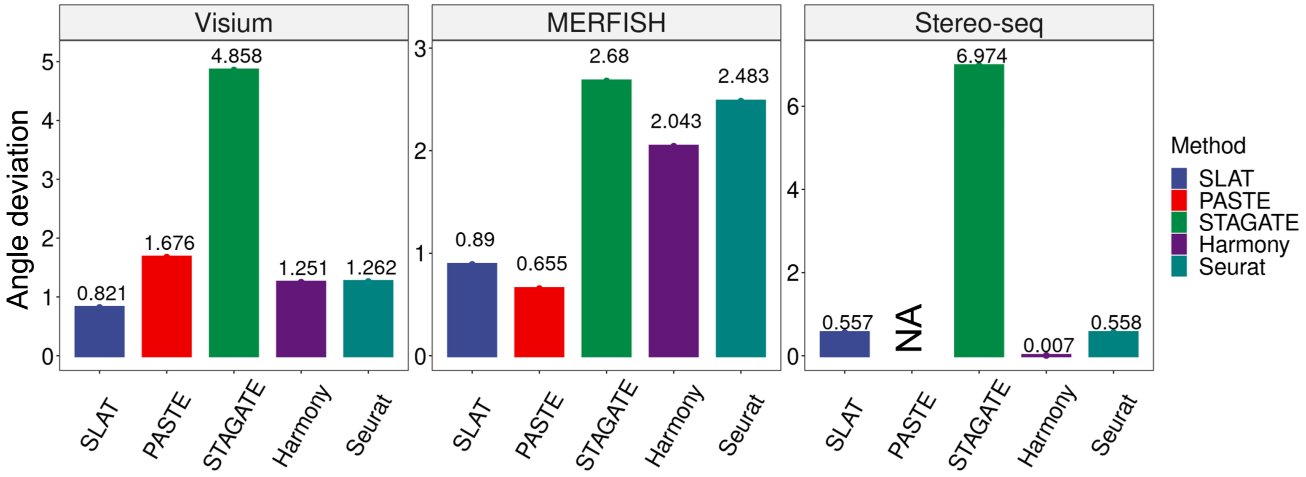

Supplementary Fig. 9 Deviation of the estimated corrective rotation angle compared to ground truth.

Angles are reported in degrees. n = 8 repeats with different random seeds. Error bars indicate mean ± s.d. Source data are provided as a Source Data file.

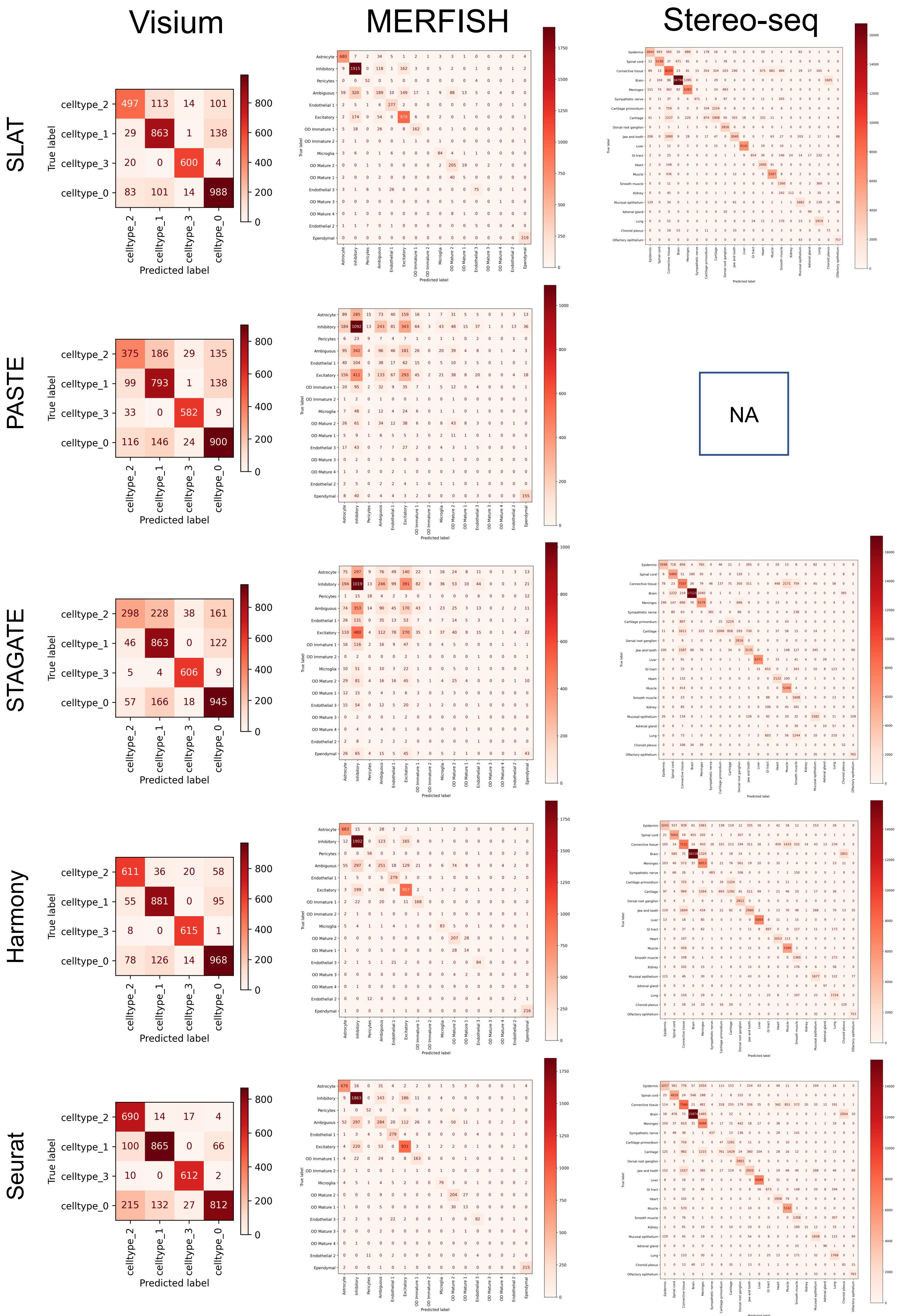

Supplementary Fig. 10 Confusion matrix of cell type matching for different methods. Source data are provided as a Source Data file.

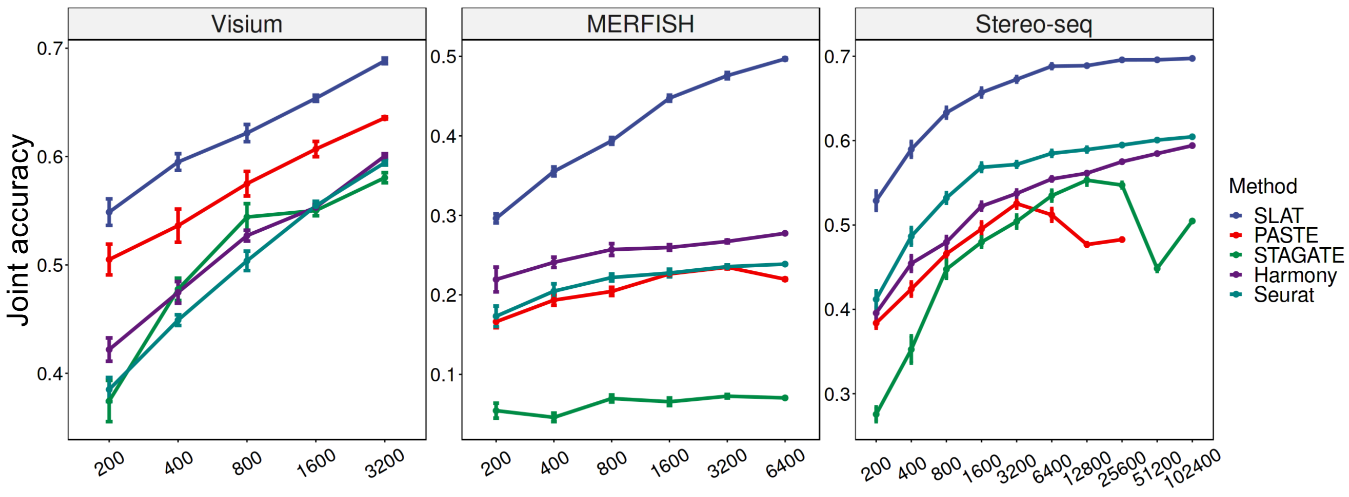

Supplementary Fig. 11 Joint accuracy in the three dataset reported in Fig. 2 with different subsampling sizes.

n = 8 repeats with different subsampling random seeds. Error bars indicate mean ± s.d. Source data are provided as a Source Data file.

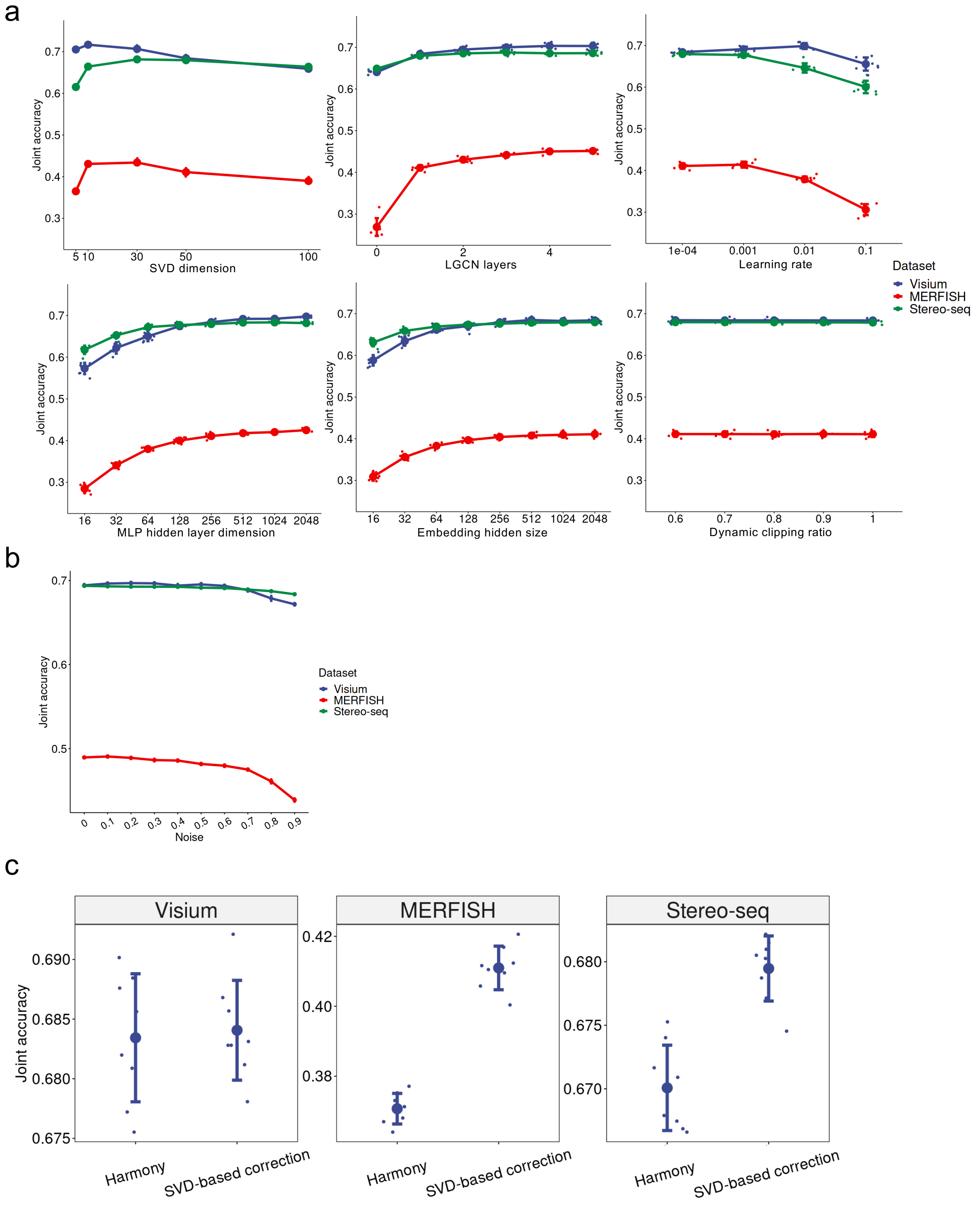

Supplementary Fig. 12 Effect of hyperparameter settings on alignment performance. **a**, Joint accuracy, which is defined as the proportion of aligned cells with both cell type and spatial region matched correctly (Methods) for SLAT with different hyperparameter settings including: SVD dimension (M), number of LGCN layers (L), learning rate, MLP hidden layer dimension, embedding hidden size (P) and dynamic clipping ratio (c). n = 8 repeats with different model random seeds. See **Methods** for detailed explanations of the hyperparameters. **b**, Changes in joint accuracy at different spatial graph corruption rates. n = 8 repeats with different model random seeds. **c**, Comparison of different batch correction modules of SLAT. n = 8 repeats with different random seeds. Error bars indicate mean ± s.d. Source data are provided as a Source Data file.

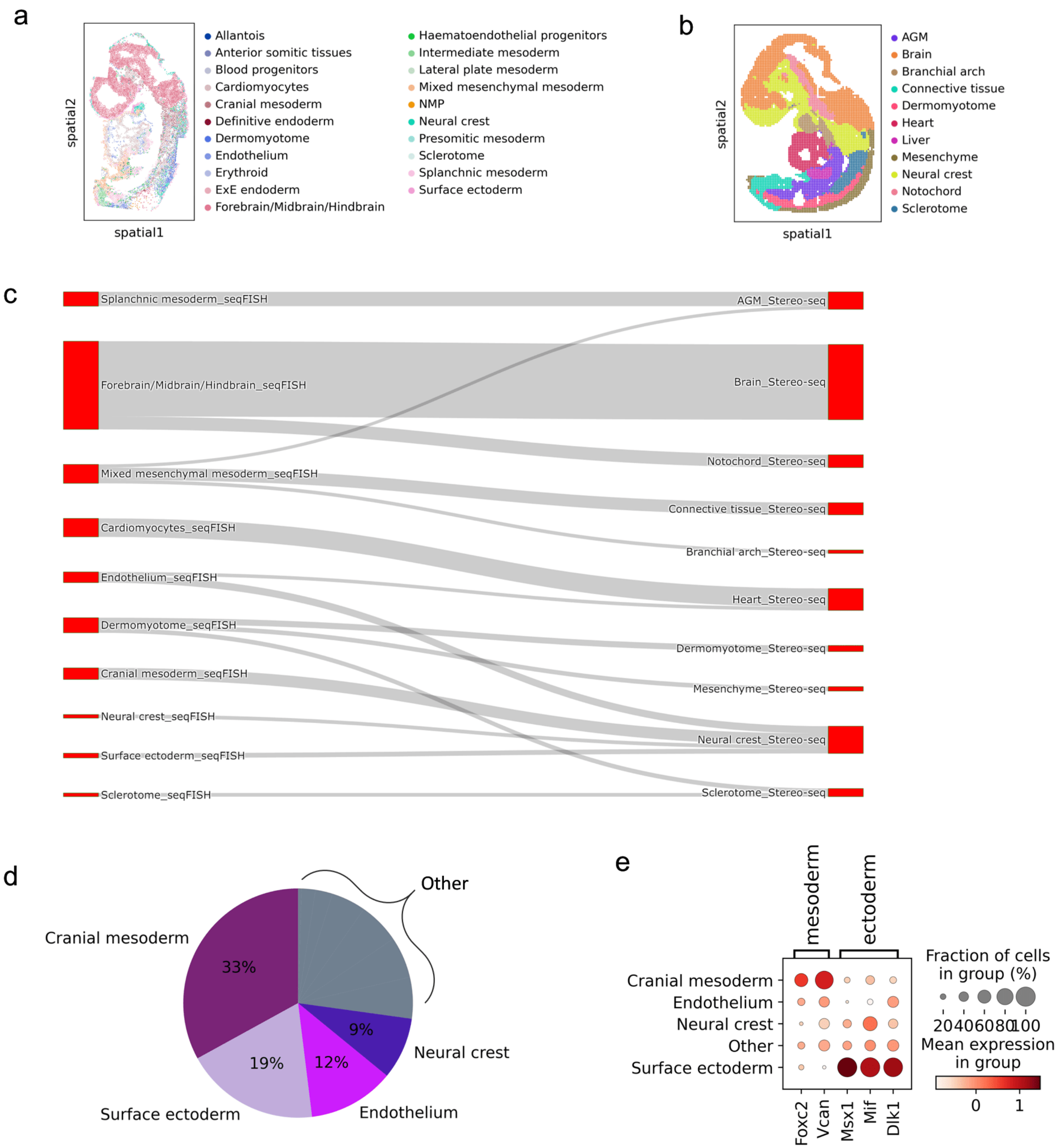

Supplementary Fig. 13 Refining cell type annotation in Stereo-seq via label transfer. **a-b**, Original annotation of **a**, the seqFISH dataset and **b**, the Stereo-seq dataset. **c**, Sanky plot showing cell type correspondence of SLAT alignment between seqFISH and Stereo-seq dataset. **d**, Proportions seqFISH transferred cell types in Stereo-seq cells originally labeled as “Neural crest”. All other cell types with proportions of less than 5% are collectively shown as “Other”. **e**, Expression of ectoderm and mesoderm marker genes as partitioned in Fig. 3c. Source data are provided as a Source Data file.

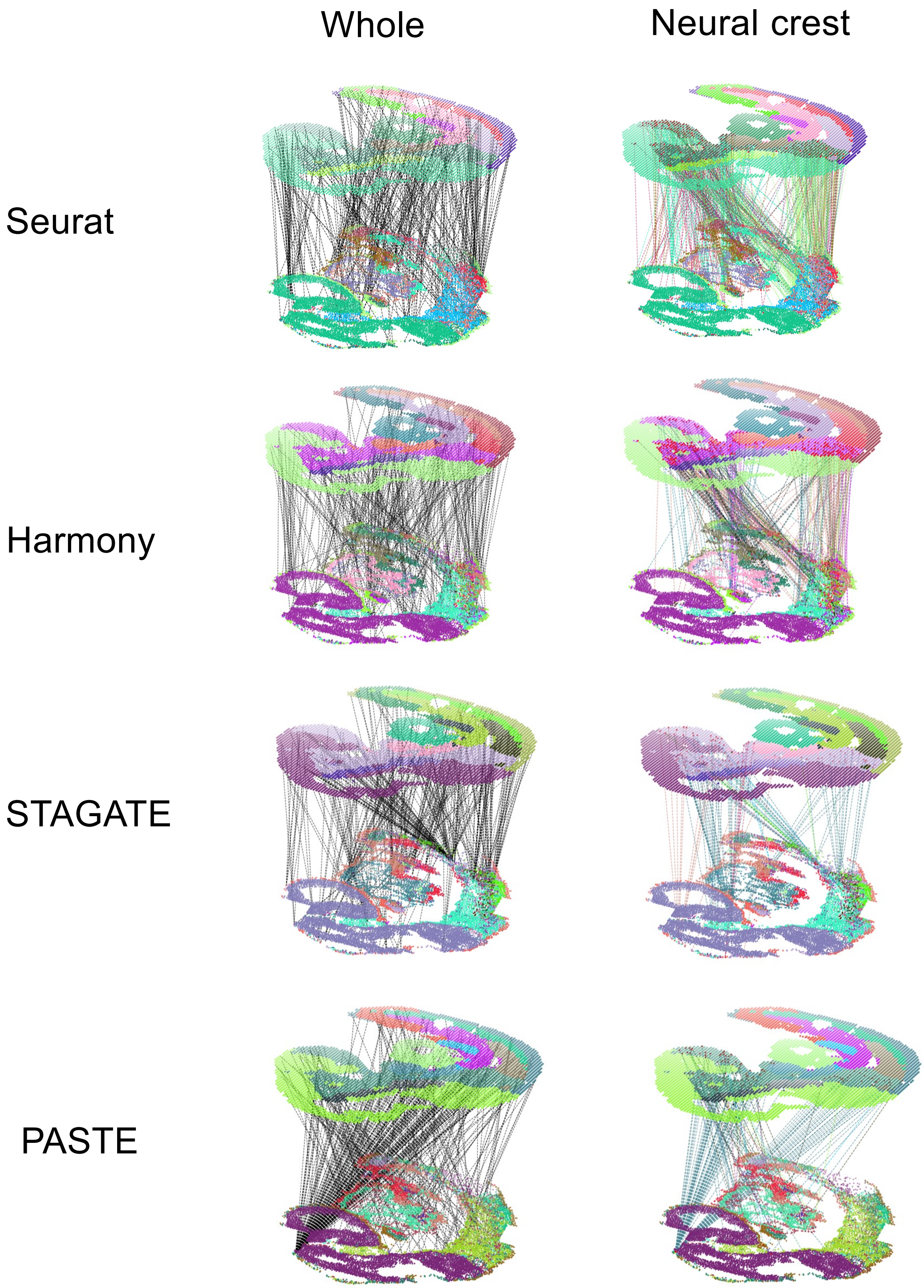

Supplementary Fig. 14 Alignment results of current methods on the seqFISH and Stereo-seq slices.

Visualization of the alignment results of seqFISH and Stereo-seq mouse embryo slices via current methods. The left panels show the complete alignment results (subsampled to 300 alignment pairs for clear visualization), while the right panels highlight cells labeled as "Neural crest" in Stereo-seq and their aligned cells in seqFISH.

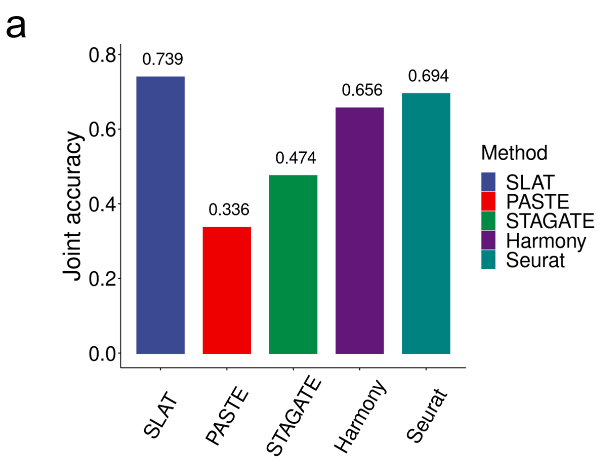

Supplementary Fig. 15 Quantitative evaluation of heterogeneous alignments.
a, Joint accuracy for seqFISH+ vs. Stereo-seq alignment. **b**, Joint accuracy for Stereo-seq vs. spatial-ATAC-seq alignment. PASTE failed due to OOM while STAGATE and Seurat CCA could not be applied because they lacked ability to handle the cross-modality alignment. Source data are provided as a Source Data file.

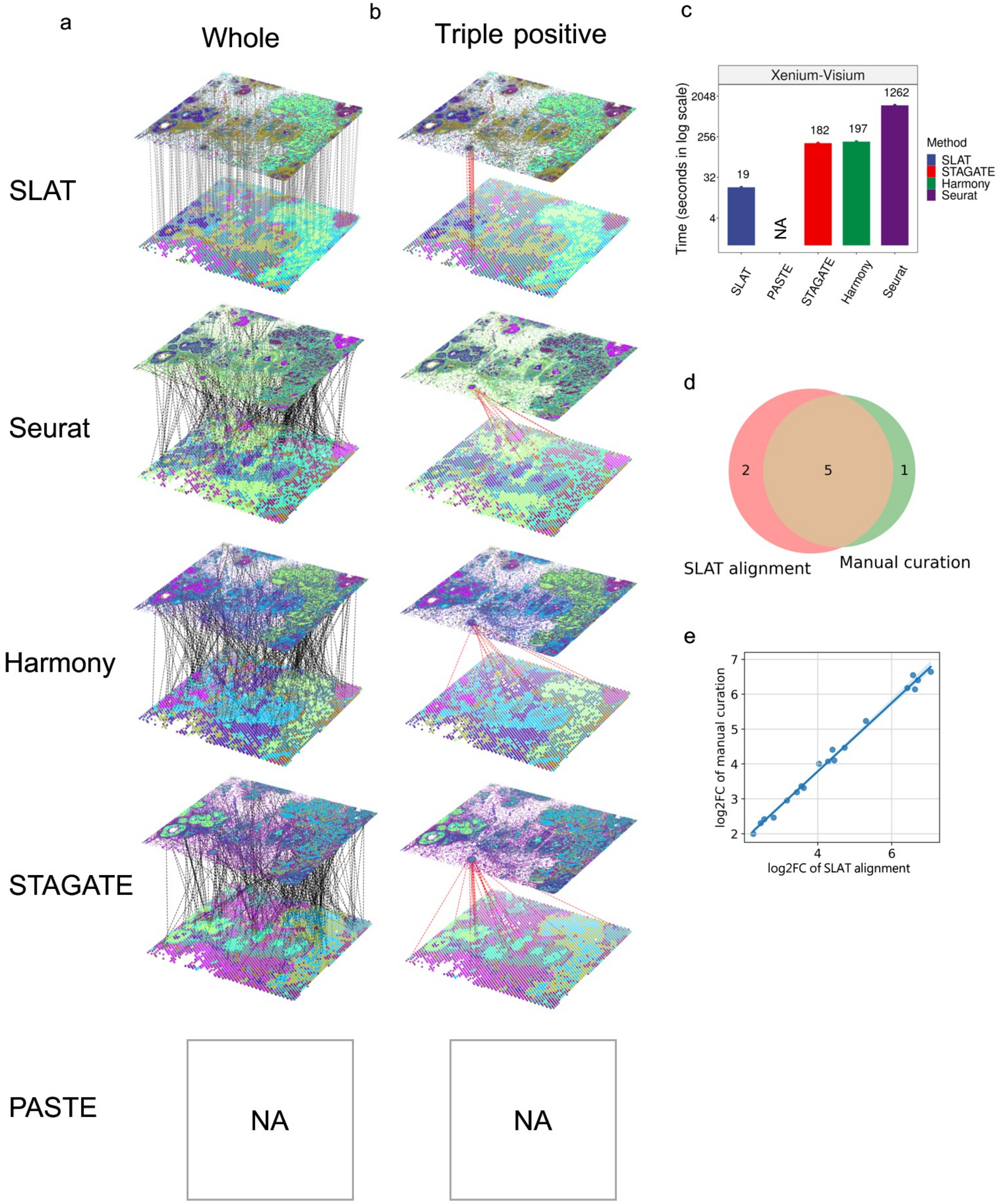

a
Whole
b
Triple positive
c
SLAT
Seurat
Harmony
STAGATE
PASTE
NA
NA
Xenium-Visium
Time (seconds in log scale)
2048
256
32
4
19
NA
182
197
1262
SLAT
PASTE
STAGATE
Harmony
Seurat
Method
SLAT
STAGATE
Harmony
Seurat
d
2
5
1
SLAT alignment
Manual curation
e
log2FC of manual curation
log2FC of SLAT alignment

Supplementary Fig. 16 Alignment results of current methods on the seqFISH and Stereo-seq slices.

Xenium and Visium are spatial technologies designed by 10x with complementary capabilities4. Xenium achieves subcellular resolution but only detects a limited set of genes, while Visium profiles the complete transcriptome with coarse spatial resolution. Specifically, the Xenium slice covers more than 100,000 cells with 313 genes detected in total, and the Visium slice contains 3,841 spots with over 20,000 genes[4]. **a,** Visualization of the alignment results of two consecutive Xenium and Visium human breast cancer slices via different methods (subsampled to 300 alignment pairs for clear visualization), **b** Highlighting cells labeled as "Triple positive breast tumor cells" (ERBB2+/ESR1+/PGR+, Supplementary Fig. 29a and 29b) in Xenium and their aligned cells in Visium. PASTE failed due to GPU memory overflow (capping at 80 G). Of note, SLAT is the only algorithm that successfully aligned Xenium-resolved rare triple positive cells to Visium slice, even though they span less than 10 spots (Supplementary Fig. 29c and 29d). **c**, Running time of different methods. **d**, Overlapping of spots between the SLAT aligned and manually curated triple positive spots. **e**, Log2 fold change of shared top 25 marker genes in e for SLAT aligned compared to manually curated triple positive spots (Supplementary Fig. 29e). Source data are provided as a Source Data file.

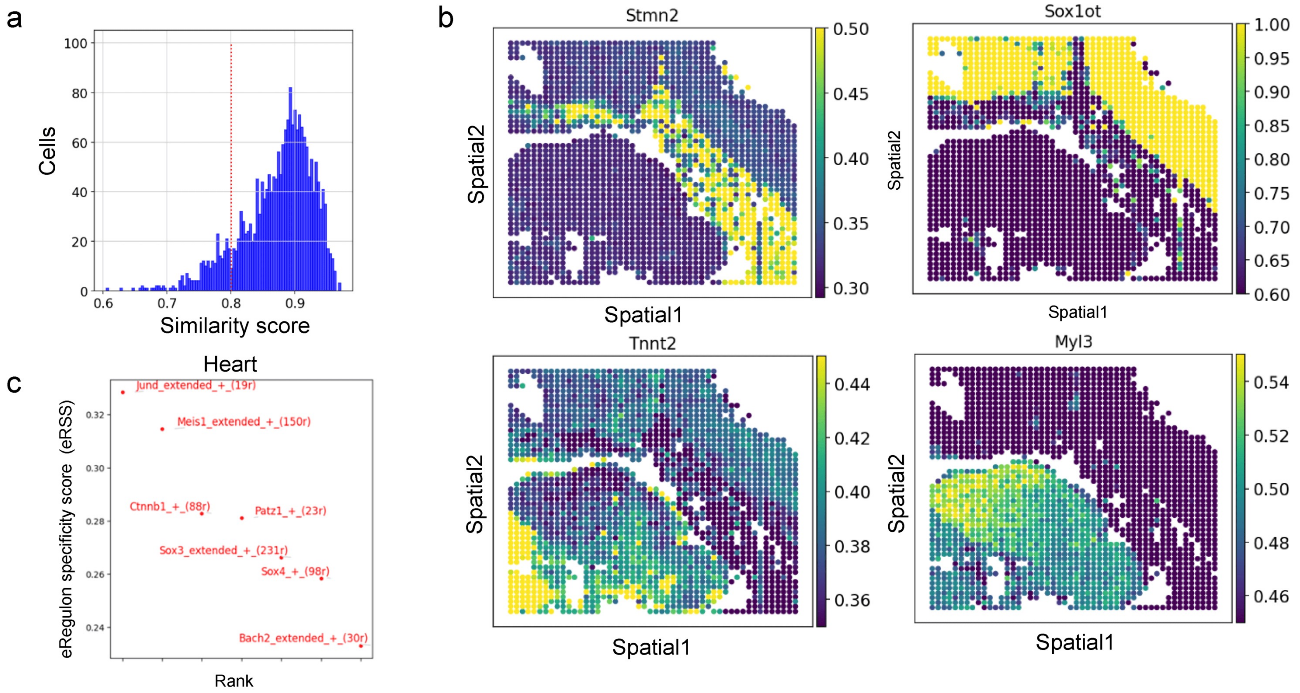

Supplementary Fig. 17 Alignment-based cross-modality annotation and regulatory reference.

a, Similarity score distribution in spatial-ATAC-seq and Stereo-seq alignment. **b**, Visualization of chromatin accessibility score of cell type marker genes in the spatial-ATAC-seq slice. **c**, Key heart regulators identified by SCENIC+ based on the SLAT alignment.

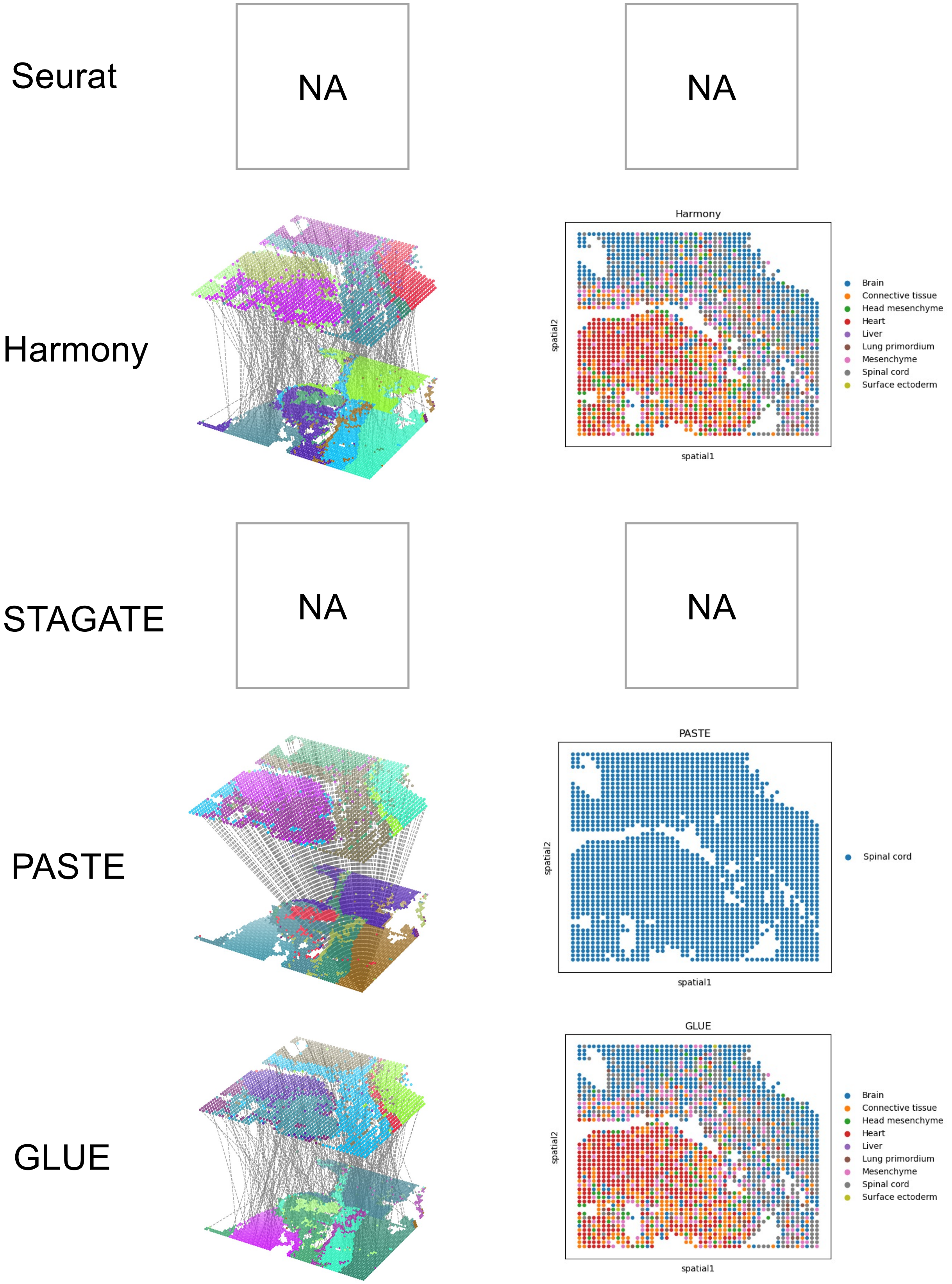

Supplementary Fig. 18 Alignment results of current methods on the spatial-ATAC-seq and Stereo-seq slices.

Visualization of the alignment results of spatial-ATAC-seq and Stereo-seq E11.5 mouse embryo slices via current methods based on GLUE embedding. The left panels show the complete alignment results (subsampled to 300 alignment pairs for clear visualization), while the right panels show cell type transfer results from Stereo-seq to spatial-ATAC-seq. Seurat and STAGATE failed because they do not support GLUE embedding as input.

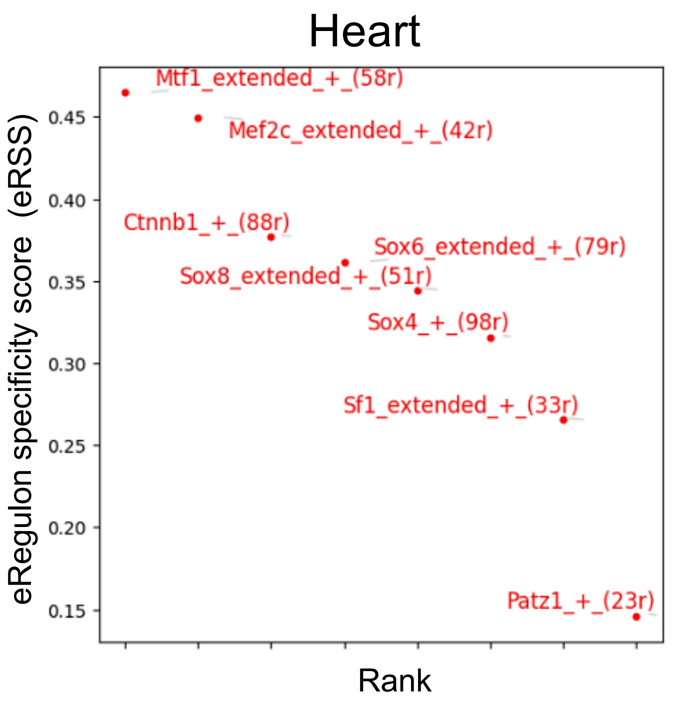

Supplementary Fig. 19 Joint regulatory inference in embryo via GLUE alignment.

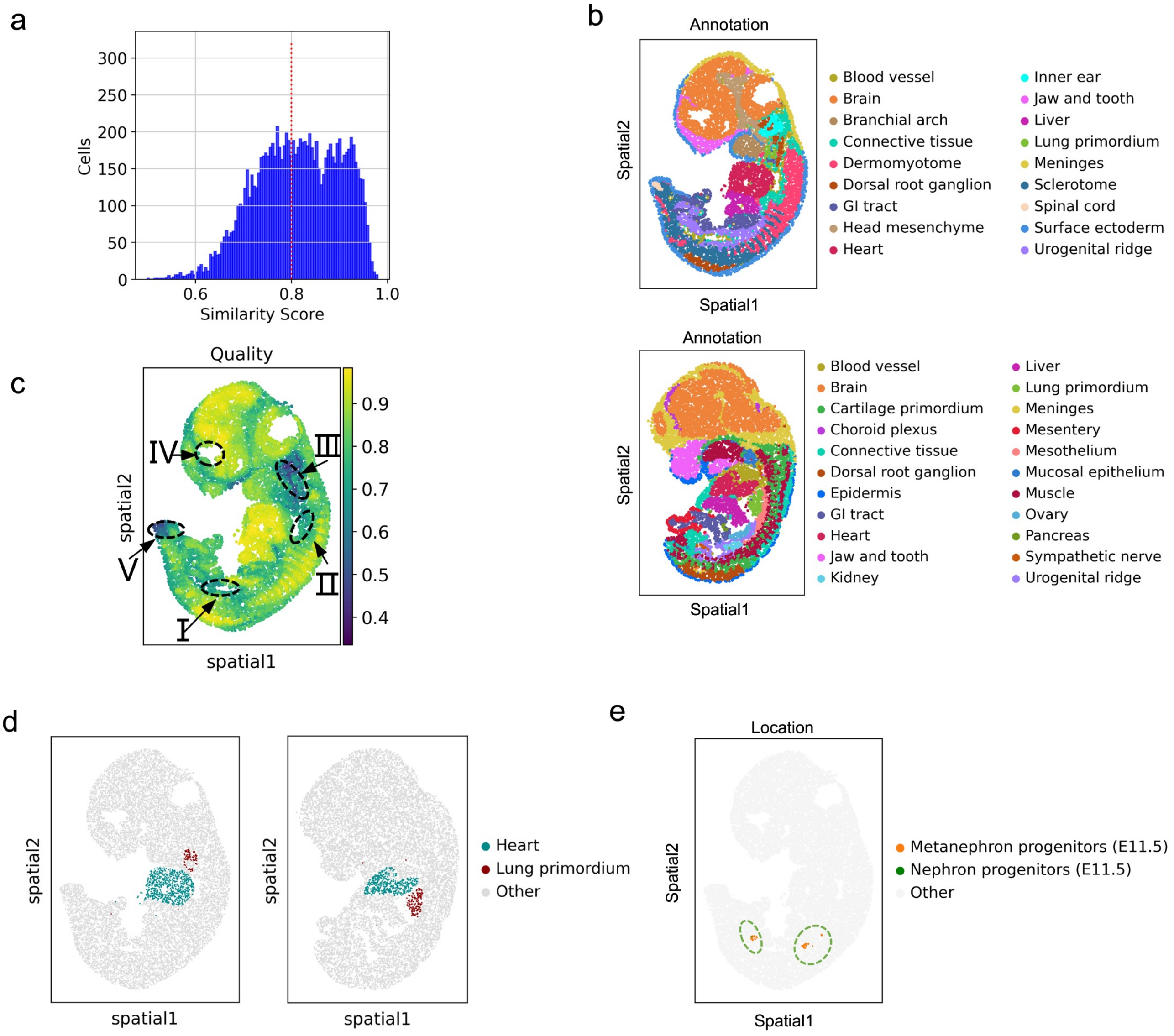

Supplementary Fig. 20 Spatial-temporal dynamics in mouse embryonic development. **a**, Similarity score distribution in E11.5 and E12.5 mouse embryo Stereo-seq slice alignment. **b**, Original annotation of E11.5 (top) and E12.5 (bottom) mouse embryo datasets. **c**, Similarity score of the E11.5 embryo. Higher scores indicate higher alignment confidence. Dashed circles highlight five regions with low similarity scores. **d**, Spatial location of “Heart” and “Lung primordium” cells in the E11.5 (left) and E12.5 (right) slice, respectively. **e**, Spatial location of “Kidney” aligned cells in E11.5, colored by cell type in Fig. 4e.

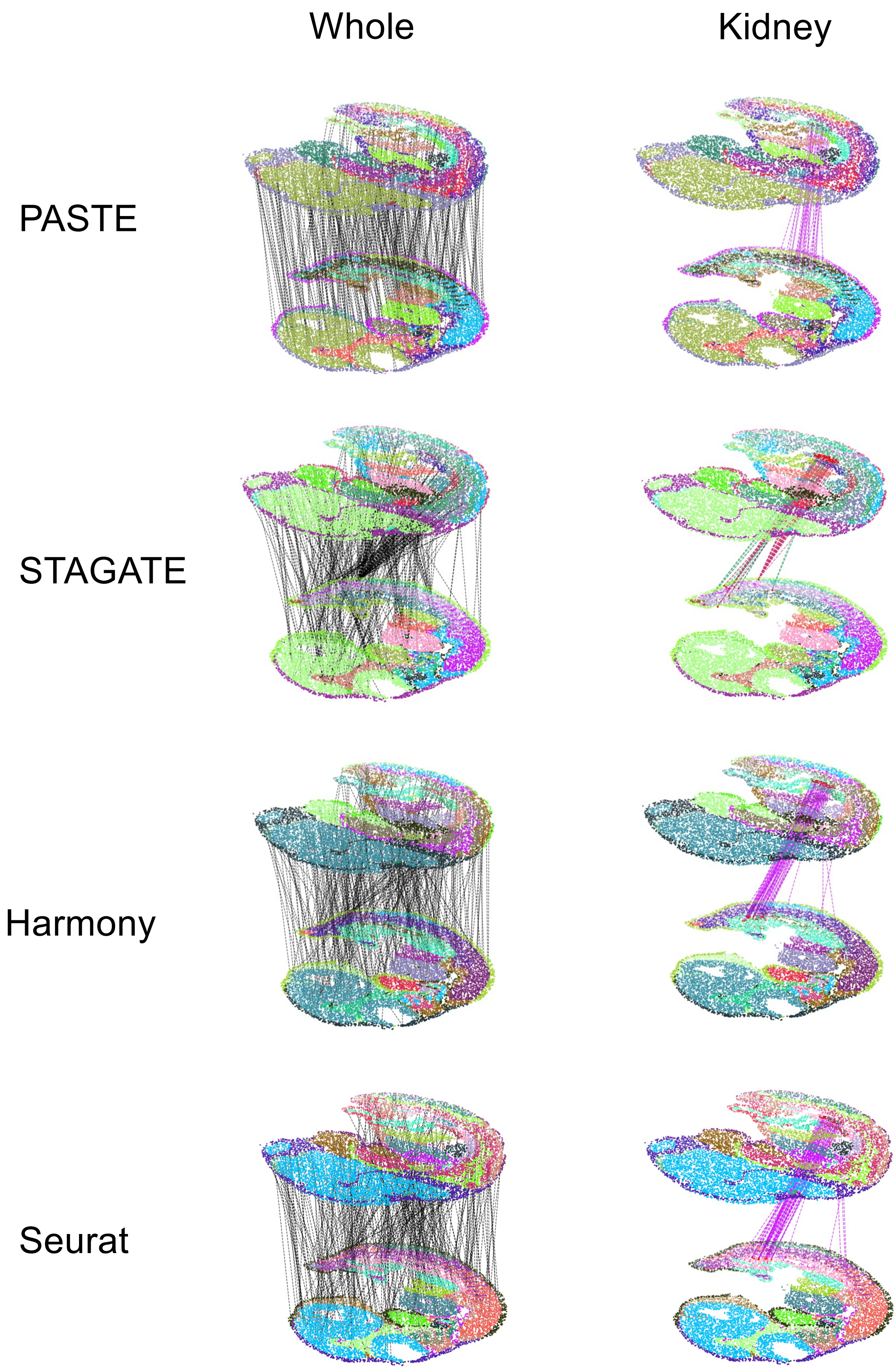

Supplementary Fig. 21 Alignment results of current methods in mouse embryonic development.

Alignment of E11.5 and E12.5 mouse embryo slices using current methods. The left panels show the complete alignments (subsampled to 300 alignment pairs for clear visualization), while the right panels highlight cells labeled as "Kidney" in E12.5 and their aligned cells in E11.5.

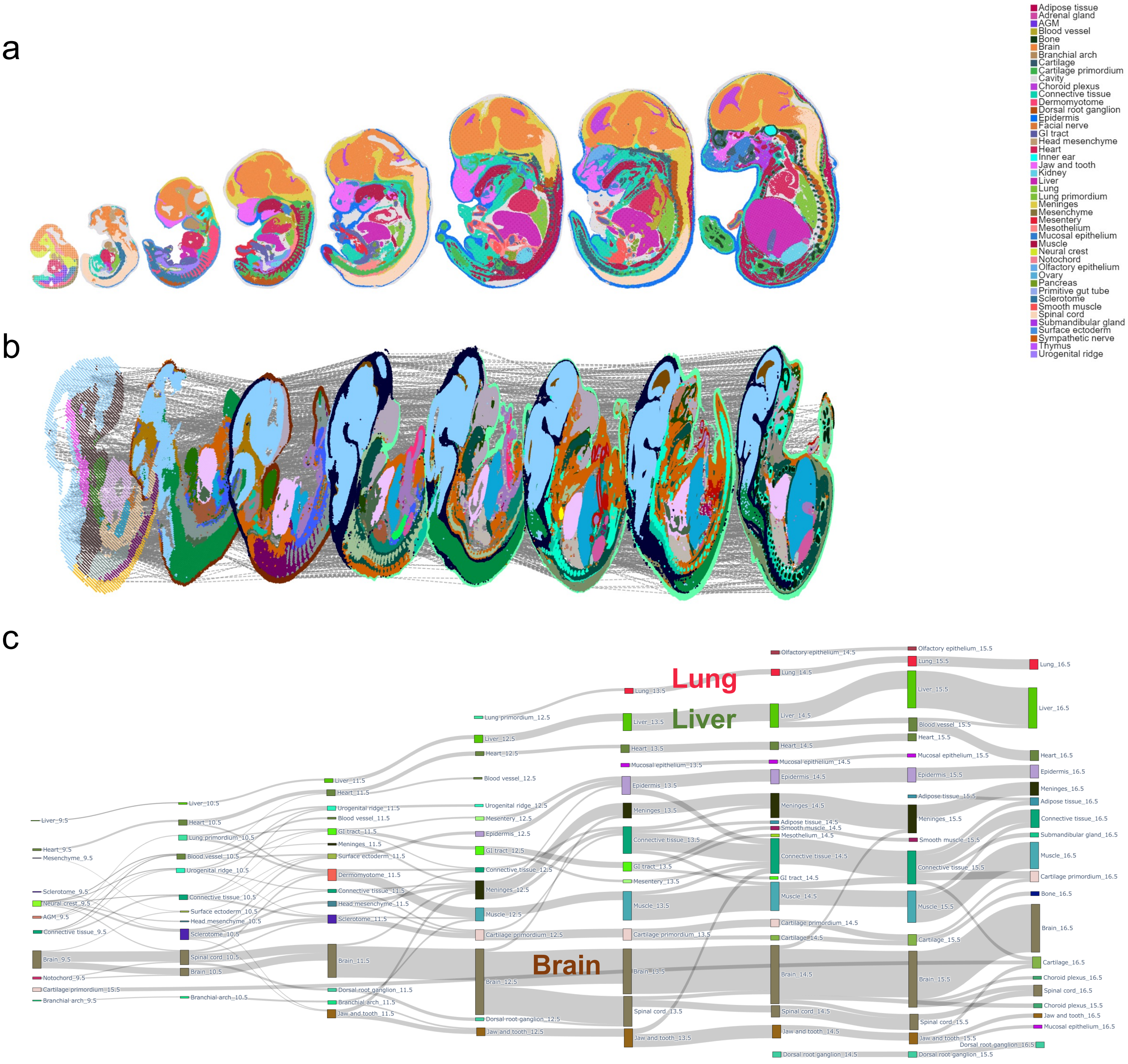

Supplementary Fig. 22 SLAT alignment of the 8 mouse embryo Stereo-seq slices. **a**, Mouse embryo Stereo-seq slices from E9.5 to E16.5. The 8 slices are temporally ordered from left to right. **b**. SLAT alignment of the 8 mouse embryo Stereo-seq slices in **a**. **c**, Sankey plot of cell type development from E9.5 to E16.5 as inferred from alignment results. Colored by the original cell type annotation. Links containing less than 1% of cells are omitted for clear visualization.

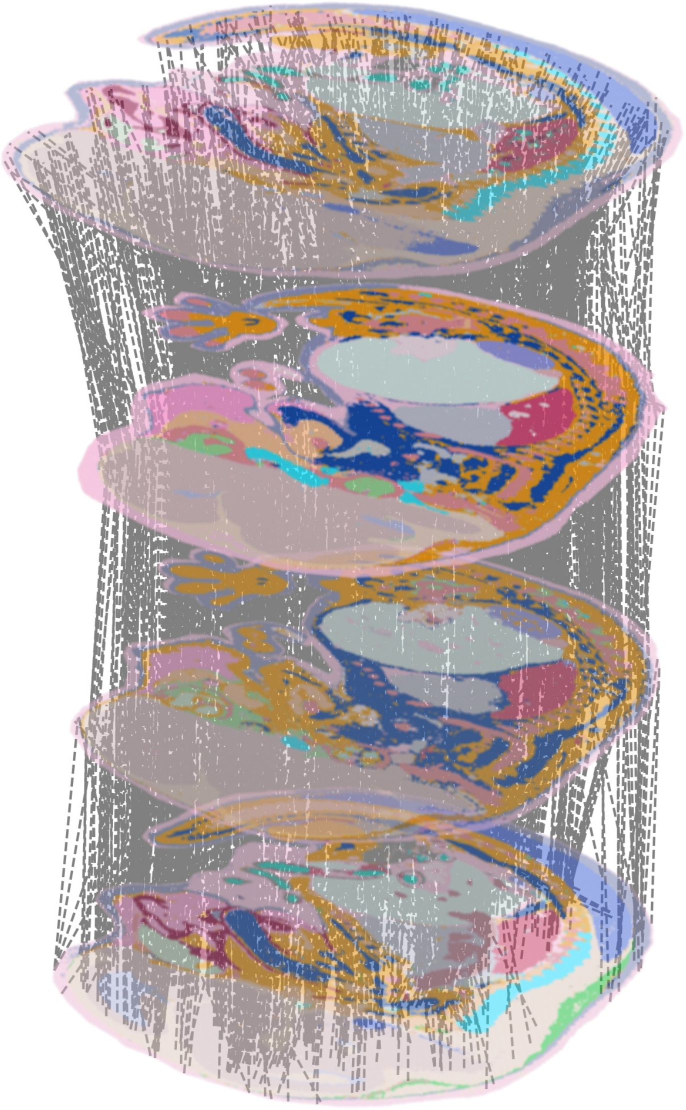

Supplementary Fig. 23 Multi-slice SLAT alignment for 3D reconstruction.
Simultaneous SLAT alignment across four slices from the same E15.5 mouse embryo, ordered by vertical position (subsampled to 300 alignment pairs for clear visualization).

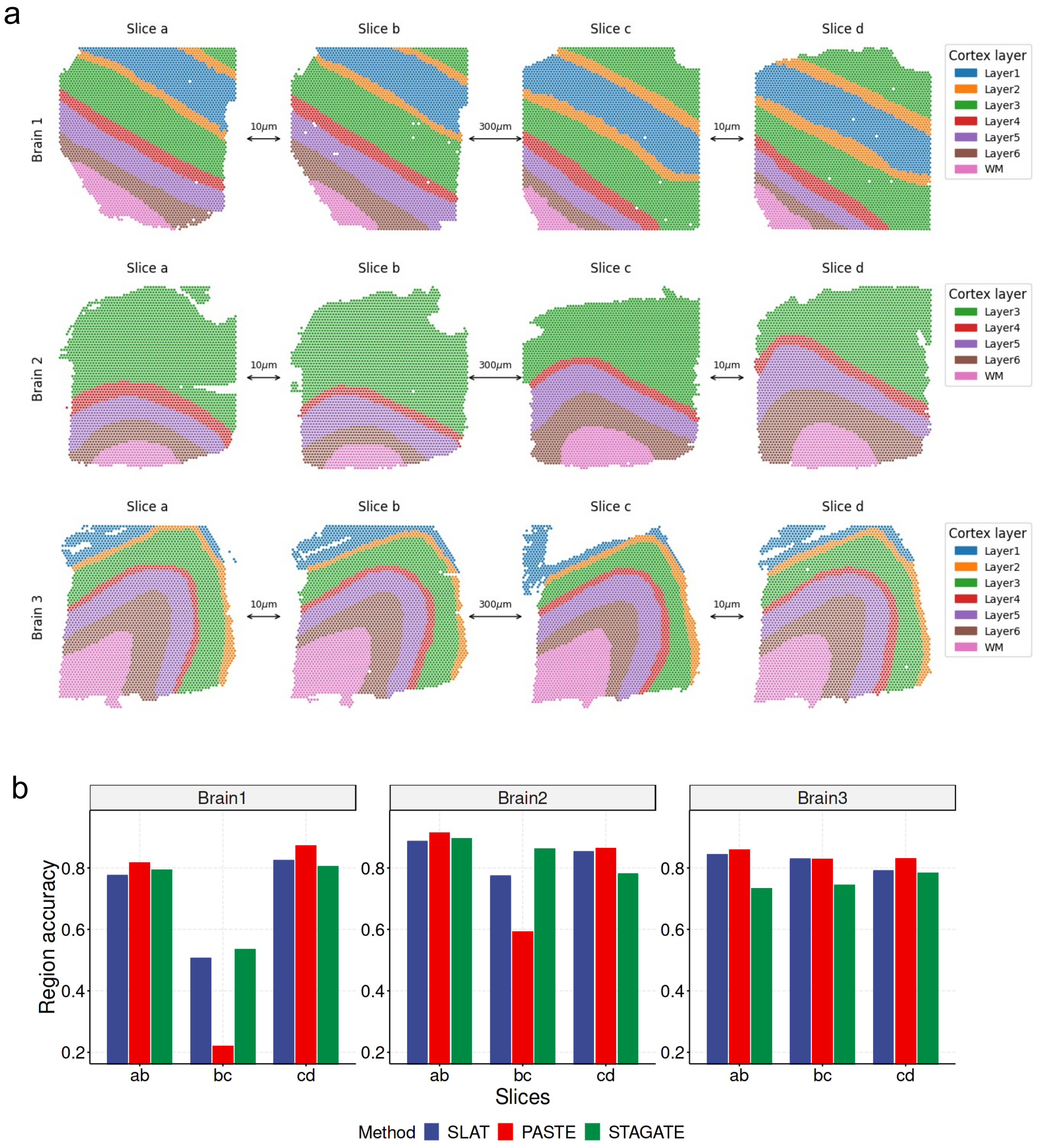

Supplementary Fig. 24 Evaluation of 3D reconstruction on consecutive brain slices. **a**, Visualization of 10x Visium slices used in 3D reconstruction benchmark (same 10x Visium datasets used in PASTE). Slices are divided into three groups, each group was consecutively sliced from the same human dorsolateral prefrontal cortex sample. The slices are colored by spatial regions annotated by original authors. **b**, Region matching accuracy[14] of different methods in 3D reconstruction. *n* = 8 repeats with different model random seeds. Dataset contains three groups, each with three consecutive slices (a, b and c, more details are in **Methods**). Source data are provided as a Source Data file.

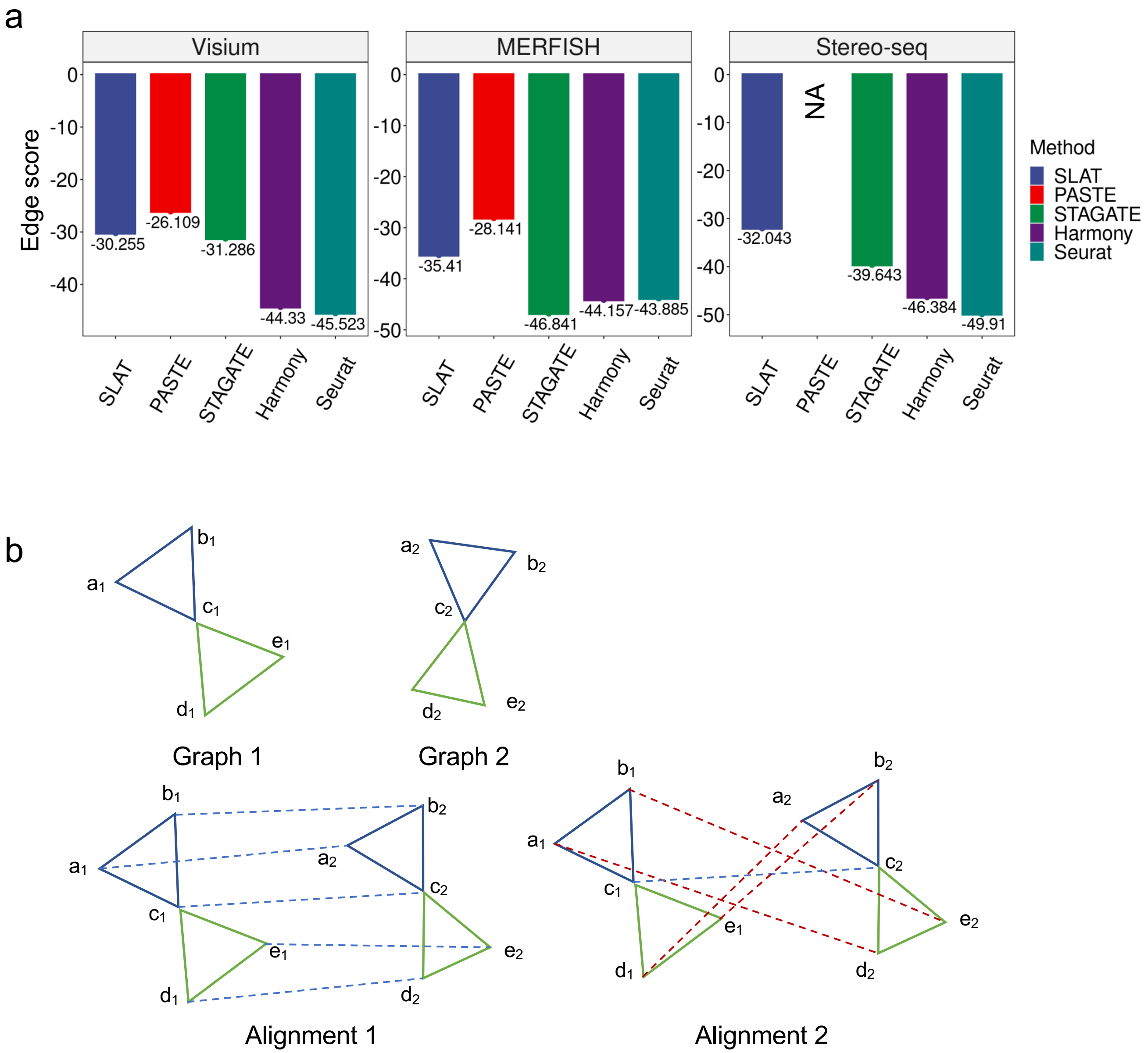

Supplementary Fig. 25 Edge score of all benchmarked methods and a counterexample. **a**, *n* = 8 repeats with different random seeds. Error bars indicate mean ± s.d. **b**, Illustration where the "edge score" metric is ineffective. "Graph 1" and "Graph 2" are two graphs with five nodes, where nodes with the same letters (e.g., a1, a2) represent ground truth node pairs. "Alignment 1" and "Alignment 2" are two graph-matchings: "Alignment 1" correctly matches all nodes between the two graphs, while only one node is correctly matched in "Alignment 2". Nevertheless, both alignments give the same "edge score". Source data are provided as a Source Data file.

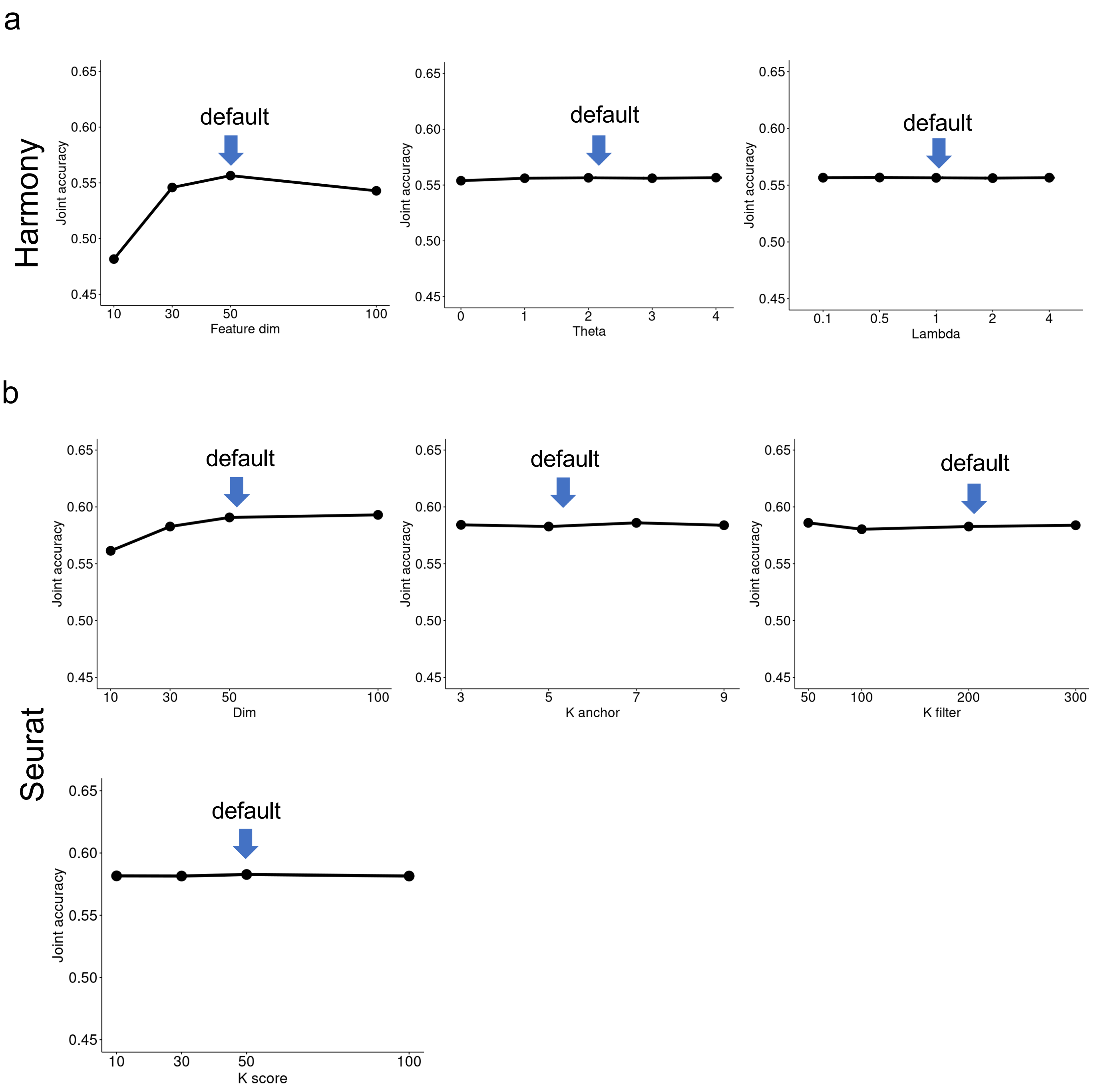
a
Harmony
default
Joint accuracy
0.65
0.60
0.55
0.50
0.45
10
30
50
100
Feature dim
0
1
2
3
4
Theta
0.1
0.5
1
2
4
Lambda
b
Seurat
Dim
3
5
7
9
K anchor
200
300
K filter
K score

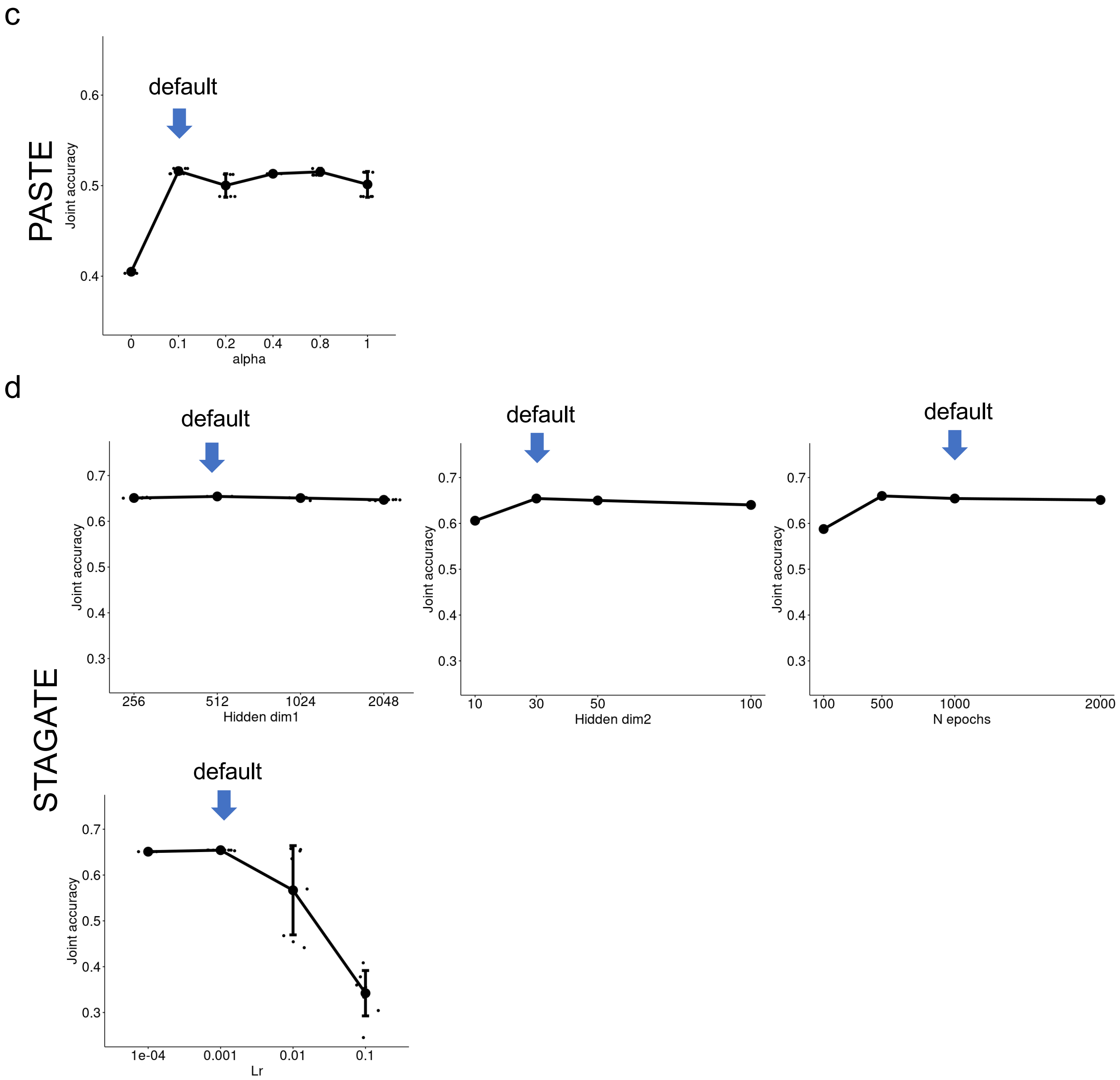

Supplementary Fig. 26 Hyperparameter search for the benchmarked methods. Hyperparameter search for Harmony (**a**), Seurat (**b**), PASTE (**c**), and STAGATE (**d**), respectively. n = 8 repeats with different random seeds. Error bars indicate mean ± s.d. Source data are provided as a Source Data file.

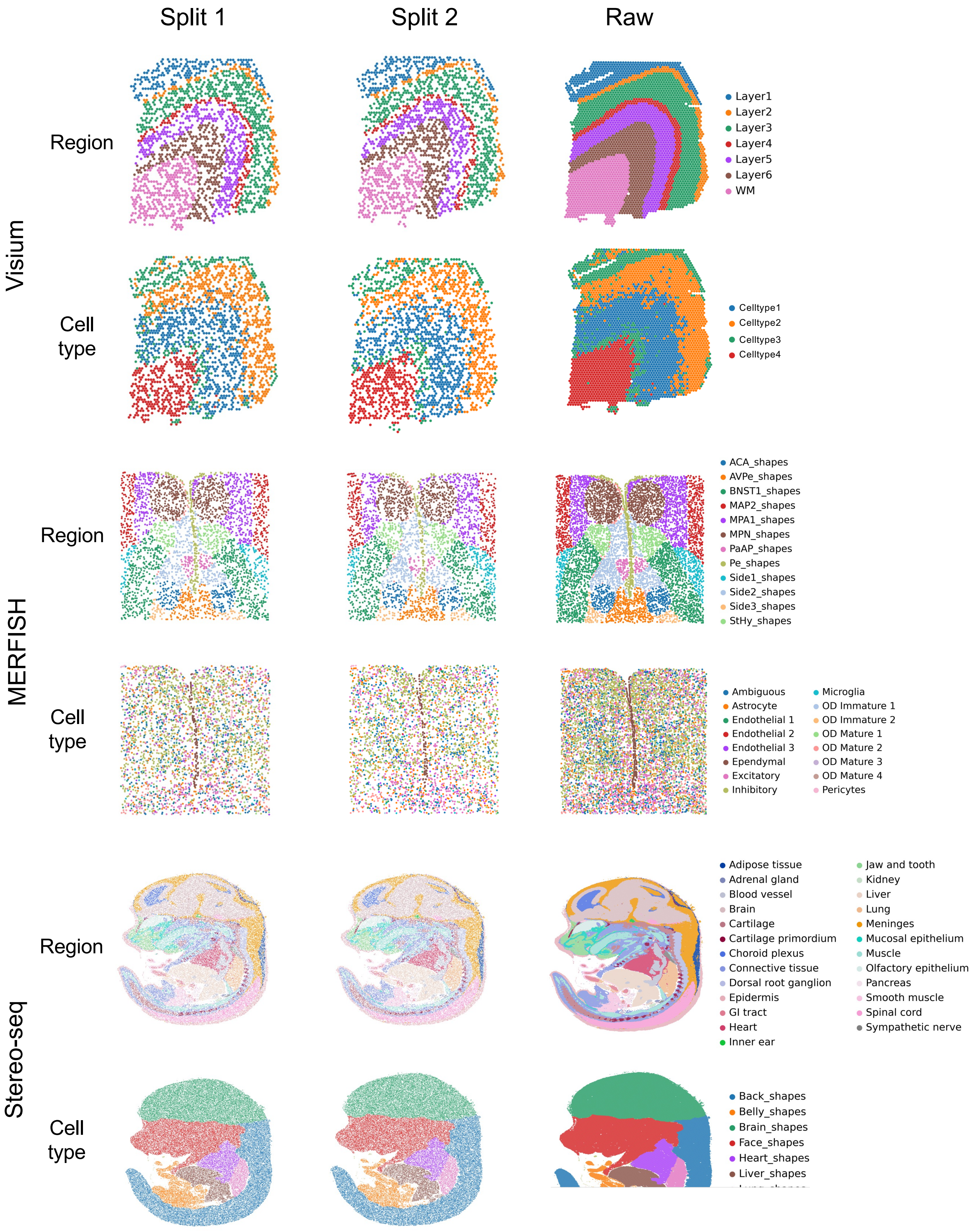

Supplementary Fig. 27 Visualization of split datasets.
Visualization of randomly split slices and raw slices used in the benchmark. Slices are colored by spatial regions and cell types, respectively.

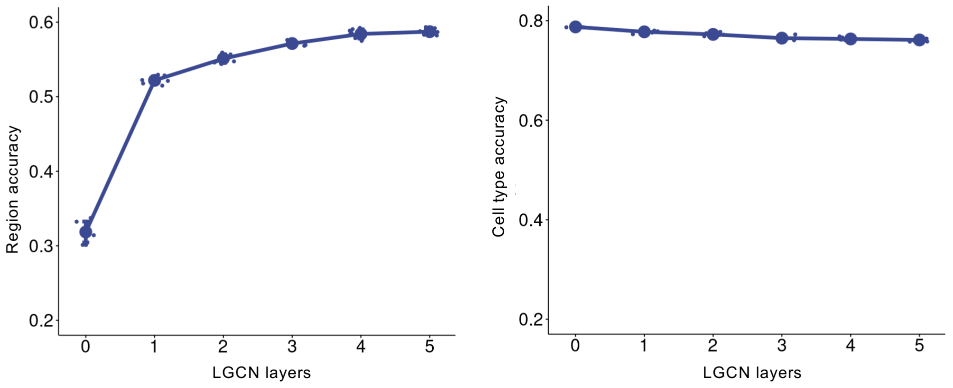

Supplementary Fig. 28 Region accuracy and cell type accuracy in MERFISH dataset with different LGCN layers.

Region accuracy (left), and cell type accuracy (right) with different LGCN layers. $n = 8$ repeats with different random seeds. Error bars indicate mean ± s.d. Source data are provided as a Source Data file.

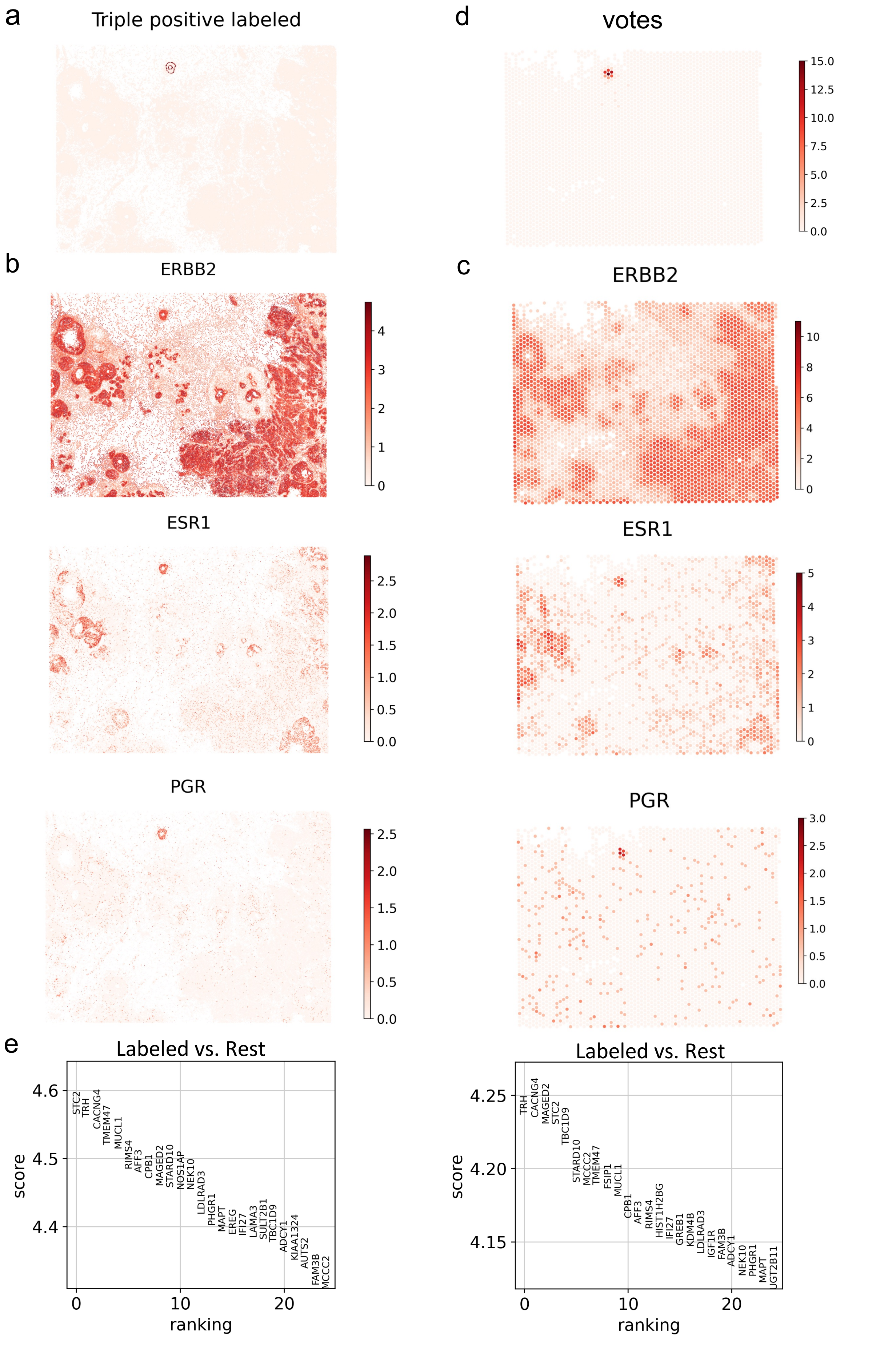

Supplementary Fig. 29 Xenium and Visium datasets.

a, Manually annotated triple positive breast tumor cells in the Xenium slice. **b**, **c**, Expression of three markers of triple positive breast cancer (*ERBB2*, *ESR1*, *PGR*) in the Visium (**b**) and Xenium (**c**) dataset. **d**, Number of Xenium cells aligned to each Visium spot. **e**, Top 25 highly expressed genes of the SLAT aligned (left panel) and manual curated (right panel) triple positive spots.

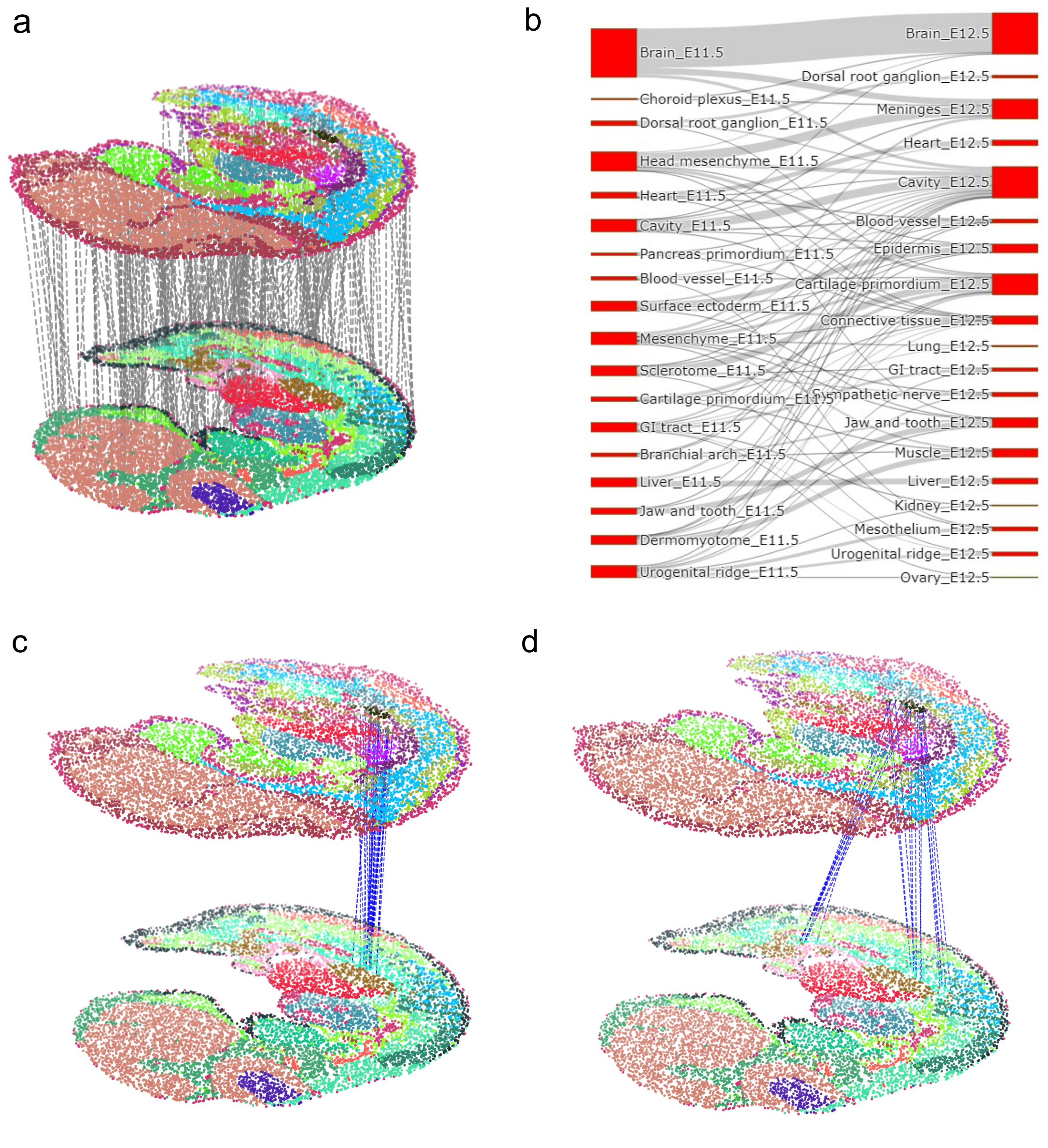

Supplementary Fig. 30 Alignment of another mouse embryo E11.5 and E12.5 slices.
a, Visualization of the alignment results of another mouse embryo E11.5 and E12.5 mouse embryo slices. **b**, Sanky plot showing cell type correspondence of SLAT alignment between the two slices. **c**, **d**, Alignment visualization highlighting cells labeled as “Kidney” (**c**) and “Ovary” (**d**) in E12.5 and their aligned cells in E11.5, respectively.

Supplementary Tables

Index	Publication	Species	Tissue	Technology	Resolution	Cells/Spots	Genes	Download Link
1	Chen et al.	Mouse	Whole embryo	Stereo-seq	0.2μm	5000-100,000	>20,000	https://db.cngb.org/stomics/mosta/download/
2	Lohoff et al.	Mouse	Whole embryo	seqFISH	subcellular	~10,000	351	https://marionilab.cruk.cam.ac.uk/SpatialMouseAtlas/
3	Deng et al.	Mouse	Whole embryo	spatial-ATAC-seq	20μm	2099	>20,000	https://www.ncbi.nlm.nih.gov/geo/query/acc.cgi?acc=GSE171943
4	Jeffrey et al.	Mouse	Brain(hypothalamic preoptic)	MERFISH	subcellular	~6,500	151	https://datadryad.org/stash/dataset/doi:10.5061/dryad.8t8s248
5	Kristen et al.	Human	Brain(dorsolateral prefrontal cortex, DLPFC)	10x Visium	50μm	~3500	>20,000	https://github.com/LieberInstitute/spatialLIBD

Supplementary Table 1 Public datasets used in this study.

Components	Joint accuracy
**Batch removal + Wasserstein discriminator**	**<u>0.444</u>**
**Batch removal step only**	0.429

Supplementary Table 2 Ablation test in the homogeneous alignment of two Stereo-seq slices.

Components	Joint accuracy
**Batch removal + Wasserstein discriminator**	**<u>0.674</u>**
**Wasserstein discriminator only**	0.659
**Batch removal step only**	0.673

Supplementary Table 3 Ablation test in the heterogeneous alignment of Stereo-seq vs. spatial-ATAC-seq.
