# Supplementary material for: Spatial-linked alignment tool (SLAT) for aligning heterogenous slices
Source: Nat Commun. 2023 Nov 9;14:7236. doi: 10.1038/s41467-023-43105-5 (PMC10636043; doi:10.1038/s41467-023-43105-5)
Supplement: Supplementary file 3 — Reporting Summary [file 41467_2023_43105_MOESM3_ESM.pdf]

nature portfolio

Corresponding author(s): Ge Gao

Last updated by author(s): Oct 10, 2023

Reporting Summary

Nature Portfolio wishes to improve the reproducibility of the work that we publish. This form provides structure for consistency and transparency in reporting. For further information on Nature Portfolio policies, see our Editorial Policies and the Editorial Policy Checklist.

Statistics

For all statistical analyses, confirm that the following items are present in the figure legend, table legend, main text, or Methods section.

n/a	Confirmed	
☐	☒	The exact sample size (*n*) for each experimental group/condition, given as a discrete number and unit of measurement
☐	☒	A statement on whether measurements were taken from distinct samples or whether the same sample was measured repeatedly
☐	☒	The statistical test(s) used AND whether they are one- or two-sided<br>*Only common tests should be described solely by name; describe more complex techniques in the Methods section.*
☒	☐	A description of all covariates tested
☐	☒	A description of any assumptions or corrections, such as tests of normality and adjustment for multiple comparisons
☐	☒	A full description of the statistical parameters including central tendency (e.g. means) or other basic estimates (e.g. regression coefficient) AND variation (e.g. standard deviation) or associated estimates of uncertainty (e.g. confidence intervals)
☐	☒	For null hypothesis testing, the test statistic (e.g. *F*, *t*, *r*) with confidence intervals, effect sizes, degrees of freedom and *P* value noted<br>*Give P values as exact values whenever suitable.*
☒	☐	For Bayesian analysis, information on the choice of priors and Markov chain Monte Carlo settings
☒	☐	For hierarchical and complex designs, identification of the appropriate level for tests and full reporting of outcomes
☐	☒	Estimates of effect sizes (e.g. Cohen's *d*, Pearson's *r*), indicating how they were calculated

Our web collection on statistics for biologists contains articles on many of the points above.

Software and code

Policy information about availability of computer code

Data collection	All data analyzed within this manuscript are publicly available. No additional software was used for the data collection process.
Data analysis	SLAT analysis was performed using Python (3.8) package scSLAT (v0.2.0) (available at https://github.com/gao-lab/SLAT). Benchmarked methods were performed using software packages Seurat (v4.1.1), harmonypy (v0.0.6), paste-bio (v1.3.0), "STAGATE_pyG" (latest commit 8b9c8ef), respectively. Benchmarking pipeline was implemented using Snakemake (v7.12.0).All benchmarked methods were allocated 16 cores of Intel Xeon Platinum 8358 CPU, 128 GB of RAM, and a NVIDIA A100 GPU with 80 GB VRAM by the Slurm workload management system. Full benchmark scripts and environment configuration also be found at https://github.com/gao-lab/SLAT.

For manuscripts utilizing custom algorithms or software that are central to the research but not yet described in published literature, software must be made available to editors and reviewers. We strongly encourage code deposition in a community repository (e.g. GitHub). See the Nature Portfolio guidelines for submitting code & software for further information.

Data

Policy information about availability of data

All manuscripts must include a data availability statement. This statement should provide the following information, where applicable:

- Accession codes, unique identifiers, or web links for publicly available datasets
- A description of any restrictions on data availability
- For clinical datasets or third party data, please ensure that the statement adheres to our policy

All datasets used in this study are already published and were obtained from public data repositories. See Supplementary Table 1 for detailed information on single

cell spatial datasets used in this study, including original papers as well as assess URLs.

Research involving human participants, their data, or biological material

Policy information about studies with human participants or human data. See also policy information about sex, gender (identity/presentation), and sexual orientation and race, ethnicity and racism.

Reporting on sex and gender	None
Reporting on race, ethnicity, or other socially relevant groupings	None
Population characteristics	None
Recruitment	None
Ethics oversight	None

Note that full information on the approval of the study protocol must also be provided in the manuscript.

Field-specific reporting

Please select the one below that is the best fit for your research. If you are not sure, read the appropriate sections before making your selection.

☒ Life sciences ☐ Behavioural & social sciences ☐ Ecological, evolutionary & environmental sciences

For a reference copy of the document with all sections, see nature.com/documents/nr-reporting-summary-flat.pdf

Life sciences study design

All studies must disclose on these points even when the disclosure is negative.

Sample size	No sample size chosen was performed. All data used in this manuscript were taken from public resources and used to demonstrate the ability of SLAT. We used all spatial slices from different technologies with different size and spatial resolution for benchmarking. The 10x Visium dataset contains 9 slices of human brain, MERFISH dataset contains 12 slices of mouse brain, Stereo-seq dataset contains 4 slices. In case studies, we use SLAT to align two slices from different technologies, different modalities, different time points, respectively.
Data exclusions	No data were excluded from analysis
Replication	All computational experiments were assembled using Snakemake and environment configuration files are provided to ensure reproducibility. All attempts at replication were successful and can be performed independently. We used n=8 repeats which was empirically sufficient to capture the mean and variance of model performance.
Randomization	Complete randomization was used for corruption and subsampling evaluations.
Blinding	All computational methods were blinded to ground truth pairing information during performance evaluation.

Reporting for specific materials, systems and methods

We require information from authors about some types of materials, experimental systems and methods used in many studies. Here, indicate whether each material, system or method listed is relevant to your study. If you are not sure if a list item applies to your research, read the appropriate section before selecting a response.

Materials & experimental systems

n/a	Involved in the study	
☒	☐	Antibodies
☒	☐	Eukaryotic cell lines
☒	☐	Palaeontology and archaeology
☒	☐	Animals and other organisms
☒	☐	Clinical data
☒	☐	Dual use research of concern
☒	☐	Plants

Methods

n/a	Involved in the study	
☒	☐	ChIP-seq
☒	☐	Flow cytometry
☒	☐	MRI-based neuroimaging
